# Shengjiang Xiexin Decoction Alters Pharmacokinetics of Irinotecan by Regulating Metabolic Enzymes and Transporters: A Multi-Target Therapy for Alleviating the Gastrointestinal Toxicity

**DOI:** 10.3389/fphar.2017.00769

**Published:** 2017-10-27

**Authors:** Huan-yu Guan, Peng-fei Li, Xiao-ming Wang, Jia-jing Yue, Yang He, Xiao-mei Luo, Mei-feng Su, Shang-gao Liao, Yue Shi

**Affiliations:** ^1^Institute of Medicinal Plant Development, Chinese Academy of Medical Sciences and Peking Union Medical College, Beijing, China; ^2^School of Pharmaceutical Sciences, Guizhou Medical University, Guiyang, China

**Keywords:** Shengjiang Xiexin decoction, irinotecan (CPT-11), diarrhea, pharmacokinetics, UHPLC-MS/MS

## Abstract

Shengjiang Xiexin decoction (SXD), a classic traditional Chinese medical formula chronicled in *Shang Han Lun*, is used in modern clinical practice to decrease gastrointestinal toxicity induced by the chemotherapeutic drug irinotecan (CPT-11). In this study, the effect of SXD on the pharmacokinetics of CPT-11 and its active metabolites (SN-38 and SN-38G), and the underlying mechanisms were further examined. An ultra-high-performance liquid chromatography-tandem mass spectrometry (UHPLC-MS/MS) method was developed and validated for the simultaneous quantification of CPT-11, SN-38, and SN-38G in the plasma, bile, liver, intestine, and intestinal contents of control and SXD-pre-treated rats after intravenous administration of CPT-11. SXD pretreatment increased the area under the curve (AUC) and the initial plasma concentration (C_0_) of CPT-11 but decreased the plasma clearance (CL). The AUC and the maximum plasma concentration (C_max_) of SN-38 decreased, whereas the C_max_ of SN-38G increased. Compared with that of the control group, the biliary excretion of CPT-11, SN-38, and SN-38G was inhibited. The CPT-11, SN-38, and SN-38G concentrations in the liver, intestine, and intestinal contents were different between the two groups. Furthermore, the hepatic expression of multidrug resistance-associated protein-2 (Mrp-2), P-glycoprotein (P-gp), and carboxylesterase 2 (CES2) was significantly down-regulated by SXD, while the hepatic and jejunal uridine diphosphate (UDP)-glucuronosyltransferase 1A1 (UGT1A1) expression was elevated. The hydrolysis of CPT-11 to SN-38 by CES and the glucuronidation of SN-38 to SN-38G by UGT were affected by liver and jejunum S9 fractions from rats pre-treated with SXD. Therefore, this study demonstrated for the first time that SXD could alter the pharmacokinetics of CPT-11 and its metabolites to alleviate CPT-11-induced diarrhea. And the underlying mechanism of drug interaction between CPT-11 and SXD involves decreasing hepatic Mrp-2 and P-gp expression and altering the activities of CES and UGT.

## Introduction

Irinotecan (CPT-11), which is a semi-synthetic derivative of camptothecin (CPT), is a topoisomerase I inhibitor used for treating colon, lung, pancreatic, cervical, and ovarian cancers (Alimonti et al., 2004). However, its efficacy and utility are compromised due to severe delayed-onset diarrhea caused by CPT-11 bioactivation and subsequent metabolism. The metabolism of CPT-11 is complex and involves a number of enzymes and transporters (Smith et al., 2006; Swami et al., 2013). As a pro-drug, CPT-11 undergoes hydrolysis mediated by carboxylesterase (CES) into its active metabolite, SN-38, primarily in the liver. SN-38 is considered to be responsible for the induction of diarrhea (Swami et al., 2013). SN-38 is inactivated and detoxified to SN-38 glucuronide (SN-38G) primarily through glucuronidation by hepatic uridine diphosphate (UDP)-glucuronosyltransferase 1A1 (UGT1A1). Most of CPT-11 and its metabolites are excreted into the intestinal lumen *via* bile, primarily mediated by multidrug resistance-associated protein-2 (MRP-2), breast cancer resistance protein (Bcrp), and P-glycoprotein (P-gp) (Yang et al., 2005; Bansal et al., 2009; Yokooji et al., 2013). The SN-38G that is excreted *via* bile is deconjugated by bacterial β-glucuronidase to SN-38, leading to the accumulation of SN-38 in the intestine. Both SN-38 and CPT-11 can be reabsorbed into the enterohepatic circulation (Yang et al., 2005).

To date, various prophylactic and curative strategies have been used to prevent and alleviate the diarrhea induced by CPT-11, including transporter inhibition [e.g., cyclosporine (Arimori et al., 2003) and probenecid (Horikawa et al., 2002a,b)] and metabolic enzyme induction and inhibition [e.g., chrysin (Tobin et al., 2006), neomycin (Kehrer et al., 2001), and sulfonamide derivatives (Wadkins et al., 2004)]. However, since the pathogenesis of CPT-11-induced diarrhea involves multiple factors and steps, most strategies designed to act against individual targets could not completely block the delayed-onset diarrhea. Hence, the use of multi-compound multi-target therapy (Wang et al., 2012) to control gastrointestinal toxicity while maintaining the anticancer efficacy of CPT-11 has attracted substantial attention. Various traditional Chinese medicines (TCMs) have been used in clinical practice to treat cancer-related symptoms or reduce chemotherapy-associated toxicity for thousands of years. These TCM formulae, which have therapeutic effects supported by broad clinical practice, represent valuable sources for the development of multi-compound multi-target therapies to control gastrointestinal toxicity.

Shengjiang Xiexin decoction (SXD), as chronicled in *Shang Han Lun*, is composed of *Pinellia ternata* [“banxia” in Chinese, the rhizome of *P. ternata* (Thunb.) Breit.], *Glycyrrhiza uralensis* (“gancao” in Chinese, the radix of *G. uralensis* Fisch.), *Coptis chinensis* (“huanglian” in Chinese, the rhizome of *C. chinensis* Franch.), *Ziziphus jujuba* (“dazao” in Chinese, the fruit of *Z. jujuba* Mill.), *Zingiber officinale* (“ganjiang” in Chinese, the rhizome of *Z. officinale* Rosc.), *Scutellaria baicalensis* (“huangqin” in Chinese, the radix of *S. baicalensis* Georgi.), *Codonopsis pilosula* [“dangshen” in Chinese, the radix of *C. pilosula* (Franch.) Nannf.], and *Zingiber recens* (“shengjiang” in Chinese, the rhizome of *Z. recens*). SXD is a classic TCM formula and has been applied to treat gastroenteritis, ulcerative colitis, and diarrhea (Guan et al., 2017). In modern clinical practice, SXD is used to alleviate CPT-11-induced gastrointestinal toxicity to avoid the incidence of diarrhea (Wang et al., 2015). SXD inhibits intestinal apoptosis and promotes the proliferation of intestinal cells (Deng et al., 2017). As a β-glucuronidase inhibitor (Narita et al., 1993), baicalin, the primary flavonoid in SXD, was deemed responsible for the reduction in CPT-11-induced gastrointestinal toxicity *via* the inhibition of the conversion of SN-38G to SN-38 in the intestinal lumen.

In our previous study, SXD was found to increase the UGT1A1 and IL-15 levels in the serum and liver homogenates from mice with colorectal carcinoma treated with CPT-11 (Peng et al., 2017). Moreover, a Banxia Xiexin decoction, which is analogous to SXD, was observed to alter the plasma pharmacokinetics of CPT-11 and decrease the plasma level of SN-38 (Shi et al., 2015). To further investigate the mechanism of action of SXD on the gastrointestinal toxicity induced by CPT-11, a sensitive and accurate ultra-high-performance liquid chromatography-electrospray ionization-tandem mass spectrometry (UHPLC-ESI-MS/MS) method was developed and validated for the simultaneous quantification of CPT-11, SN-38, and SN-38G in Sprague-Dawley (SD) rat plasma, bile, liver and intestine and intestinal contents. Using the developed method, the effects of SXD on the rat plasma concentrations, biliary excretion and tissue disposition of CPT-11 and its metabolites were evaluated *in vivo*. The results showed that SXD pretreatment could alter the pharmacokinetics of CPT-11, SN-38, and SN-38G after the intravenous (i.v.) administration of CPT-11, leading to reduced gastrointestinal toxicity. A further investigation into the mechanism of action revealed that the reduced toxicity might be related to the alteration of the activity/expression of metabolic enzymes (CES and UDP-glucuronosyltransferase) and transporters (Mrp-2 and P-glycoprotein).

## Materials and methods

### Materials and reagents

A reference standard, irinotecan hydrochloride, was purchased from the National Institute for the Control of Pharmaceutical and Biological Products (Beijing, China). CPT (purity ≥ 98%), which was used as the ISTD, was purchased from Shanghai Yuanye Biological Technology Co. Ltd. (Shanghai, China). SN-38 and SN-38 glucuronide (purity ≥ 98%) were purchased from Toronto Research Chemicals Inc. (Toronto, Canada). Irinotecan hydrochloride for injection was obtained from Hengrui Medicine Co. Ltd. (Jiangsu, China). Sterilized saline for injection was purchased from Shijiazhuang No. 4 Pharmaceutical Co. Ltd. (Hebei, China). D-saccharic acid 1, 4-lactone, alamethicin, and uridine 5′-diphosphoglucuronic acid (UDPGA) triammonium salt were purchased from Sigma-Aldrich (St. Louis, MO, USA).

HPLC-grade acetonitrile and methanol were obtained from Honeywell Burdick & Jackson Company (Mexico City, Mexico). Formic acid (HPLC grade) was purchased from ROE Scientific Inc. (Beijing, China). Deionized water for the HPLC analysis was prepared using a Milli-Q water purification system (Milford, MA, USA). All of the other reagents were of analytical grade.

### Composition and preparation of SXD

*P. ternata, G. uralensis, C. chinensis, Z. jujuba, Z. officinale, S. baicalensis, C. pilosula*, and *Z. recens* were purchased from the Huamiao Traditional Chinese Medicine Engineering Technology Development Center (Beijing, China). These samples were identified as the dried rhizome of *P. ternata* (Thunb.) Breit (processed with alumen as adjuvant material), dried root and rhizome of *G. uralensis* Fisch., dried rhizome of *C. chinensis* Franch, dried ripe fruit of *Z. jujuba* Mill., dried rhizome of *Z. officinale* Rosc., dried root of *S. baicalensis* Georgi, dried root of *C. pilosula* (Franch.) Nannf. and rhizome of *Z. recens*, respectively. A mixture of the eight crude herbal drugs was prepared in a 9:9:3:12:3:9:9:12 ratio on a dry weight basis according to the original record in *Shang Han Lun*, and the mixture was immersed in distilled water for 30 min. Subsequently, it was added to a 10-fold volume of water and then decocted twice by boiling for 1 h. The decoctions were filtered to remove the herbal residue, combined, and concentrated to generate an extract.

### Animal studies

#### Animals

SD rats (weighing 200 ± 20 g) were obtained from the Department of Laboratory Animal Resources at the National Institutes for Food and Drug Control (Beijing, China). The rats were kept under a standard 12/12 h-light/dark cycle at 20–25°C and 40–60% humidity with free access to water and a normal diet for 1 week to allow them to adapt to the environment prior to the experiment. All of the experiments were performed according to the National Institutes of Health Guidelines for Animal Research and approved by the Ethics Committee of the Institute of Medicinal Plant Development, CAMS & PUMC.

### Pharmacokinetics and tissue distributions

Twenty-four SD rats were randomly divided into four groups with six rats in each group. SXD extract (10 g/kg body weight through oral administration, 10 mL/kg) was given to the rats in groups 1 and 2 twice per day for 5 days, starting from day 0 to 1 h before CPT-11 administration. The animals in groups 3 and 4 received a blank vehicle without SXD extract in a similar fashion. At day 5, CPT-11 diluted with saline was administered intravenously to the rats *via* their tail veins at a dose of 20 mg/kg (4 mL/kg).

For the pharmacokinetics study, blood samples from groups 1 and 3 were collected at 0.083, 0.25, 0.5, 1, 2, 4, 6, 10, and 24 h post-dosing from the retro-orbital plexus into heparinized tubes. Plasma was obtained by centrifugation at 3,000 rpm for 10 min. The liver, duodenum, jejunum, ileum, cecum, colon and rectum were removed immediately. The livers were washed with cold physiological saline to remove the blood. The intestinal contents of the corresponding segments were extruded into polypropylene tubes. The intestinal segments were then opened longitudinally, gently rinsed with saline to remove the residual contents and blot-dried with neutral filter paper. All of the obtained samples were stored at −80°C until analysis.

To further measure the concentrations in the liver and intestinal tissues, the animals in groups 2 and 4 were killed by cervical dislocation at 8 h after CPT-11 i.v. administration. Their livers and intestinal segments were removed immediately. The samples were treated in the same way as those obtained at 24 h.

### Biliary excretions

Another 12 SD rats were randomly divided into two groups—an SXD-pre-treated group and a control group—with six rats in each group. SXD extract (10 g/kg, 10 mL/kg) was given to the rats in the pre-treated group twice per day for 5 days. The animals in the control groups received the corresponding volume of blank vehicle. All of the rats were anesthetized with 10% (w/v) chloral hydrate solution (3 mL/kg, i.p.) and placed under a thermo-controlled surgery platform to maintain their body temperatures. Before the CPT-11 administration and bile sample collection, the rats received surgery for the bile cannulation experiments, as described previously (Bansal et al., 2009). Bile was collected at intervals of 0–1, 1–2, 2–3, 3–4, 4–5, 5–6, 6–7, and 7–8 h after CPT-11 i.v. administration at a dose of 20 mg/kg. The samples were stored at −80°C until analysis.

### *In Vitro* incubation

The liver and jejunum S9 fractions were prepared from rats with or without SXD treatment for 5 days using a method adopted from the literature with minor modifications (Zhu et al., 2010). The livers were perfused with ice-cold 10 mM sodium phosphate buffer (pH 7.4) and chopped into tiny pieces. The jejunums were removed, washed with ice-cold 10 mM sodium phosphate buffer (pH 7.4), blot-dried with neutral filter paper, opened longitudinally and scraped with a glass slide to collect the epithelium at 4°C. The chopped livers and scraped epithelium were homogenized with three-fold volumes of ice-cold homogenization buffer [50 mM potassium phosphate buffer, pH 7.4, 250 mM sucrose, and 1 mM ethylenediaminetetraacetic acid (EDTA)] using an S10 electric tissue homogenizer and centrifuged at 9,357 rpm for 15 min at 4°C. The fat layer was discarded carefully. The supernatant was collected and stored at −80°C until use. The protein concentration was determined using a bicinchoninic acid (BCA) assay kit (Thermo Scientific, Rockford, IL, USA).

For the CES activity assay, incubations of liver or jejunum S9 (protein content: 2 mg/mL) with CPT-11 at a final concentration of 9.47 μM were performed in 50 mM potassium phosphate buffer (pH 7.4) at 37°C in Eppendorf tubes for 2 h.

For the glucuronidation activity assay, SN-38 (38.23 μM), liver or jejunum S9 (final concentration: 2 mg/mL), MgCl_2_ (0.88 mM), alamethicin (0.022 mg/mL), and D-saccharic acid 1,4-lactone (4 mM) were mixed in 50 mM potassium phosphate buffer (pH 7.4). The reaction was initiated by adding UDPGA triammonium salt (3.5 mM) and allowed to proceed at 37°C for 2 h.

The hydrolysis and glucuronidation reactions were quenched by adding 400 μL of ice-cold acetonitrile-methanol (1:1, v/v) containing 50 ng/mL CPT (ISTD). After being centrifuged at 12,000 rpm for 10 min, the supernatant was dried and processed further as described for the plasma samples. A 10 μL volume of supernatant was injected into the LC/MS/MS system for SN-38 and SN-38G analyses.

### Determination of CPT-11, SN-38, and SN-38G concentrations

#### UHPLC-MS/MS

A UHPLC-MS/MS system was used to determine the concentrations of CPT-11, SN-38, and SN-38G. A Shimadzu LC-20AD series UHPLC (Shimadzu, Kyoto, Japan) consisting of two LC-20AD XR pumps, an SIL-20A XR autosampler, a DGU-20A 3R degasser and a CTO-20AC column oven was employed. The UHPLC separation was performed on an ACQUITY UPLC® BEH C18 (2.1 × 100 mm, 1.7 μm) column using a gradient elution consisting of acetonitrile (A) and 0.1% aqueous formic acid (B) as the mobile phase at a flow rate of 0.3 mL/min. The injection volume was set to 10 μL. The gradient elution program was as follows: 10–10% A at 0–0.5 min, 10–20% A at 0.5–1.0 min, 20–20% A at 1.0–1.5 min, 20–40% A at 1.5–3.0 min, 40–100% A at 3.0–4.0 min, 100–100% A at 4.0–6.0 min, 100–10% A at 6.0–6.01 min, and 10–10% A at 6.01–8.0 min.

The UHPLC instrument was coupled to an AB SCIEX Qtrap 5500 system (AB Sciex, Framingham, MA, USA) *via* a Turbo IonSpray ionization interface. The data were analyzed using Analyst Data Acquisition and Processing software (Version 1.6, AB Sciex). The optimized conditions were as follows: curtain gas (CUR), 35.0 psi; collision gas (CAD), medium; IonSpray voltage (IS), 5,500 V; source temperature, 550°C; GS1, 40 psi; and GS2, 40 psi.

### Sample preparation

A 100 μL volume of plasma was added with 10 μL of 10% acetic acid before protein precipitation with organic solvent. The liver, intestine and intestinal contents were accurately weighed and homogenized in 3–5 volumes of ice-cold 5% perchloric acid according to the concentration using a S10 electric tissue homogenizer (Ningbo, China). The homogenization was performed in an ice bath. The homogenates were centrifuged at 10,000 rpm for 10 min at 4°C.

A 50 μL volume of ISTD solution containing CPT (200 ng/mL) was evaporated to dryness by a gentle stream of nitrogen in an Eppendorf tube. The plasma, liver, intestine, and intestinal content homogenate samples (100 μL) were added into the abovementioned tubes and then extracted with 500 μL of acetonitrile. The sample was vortexed for 1.0 min and centrifuged (12,000 rpm, 4°C, 10 min). The supernatant was separated and dried under a gentle flow of nitrogen at room temperature. The residue was dissolved with 200 μL of 0.1% aqueous formic acid and loaded onto a pre-conditioned Agela Cleanert® PEP SPE column (Tianjin, China). The SPE column was then eluted with 3 mL of 0.1% aqueous formic acid and 2 mL of methanol in succession. The methanol fraction was collected and dried under a gentle flow of nitrogen at room temperature. The residue was reconstituted with 200 μL of 60% methanol in water and centrifuged again at 12,000 rpm for 10 min. The supernatant was injected into the UHPLC-MS/MS system for analysis.

A 10 μL volume of bile sample was transferred to an Eppendorf tube containing 50 μL of 2.0 μg/mL CPT and spiked with 50 μL of 0.1% acetic acid in acetonitrile. The sample was thoroughly vortexed for 1.0 min and centrifuged at 12,000 rpm for 10 min at 4°C. The supernatant was separated and dried under a gentle flow of nitrogen. The residue was reconstituted in 2,000 μL of 60% methanol in water before analysis.

### Western blot analysis

The livers and jejunum tissue were homogenized in ice-cold radio immunoprecipitation assay (RIPA) buffer [50 mM Tris HCl, 1 mM EDTA, 150 mM NaCl, 0.1% sodium dodecyl sulfate, 0.5% sodium deoxycholate, 1.0% Nonidet P-40, and phenylmethane sulfonyl fluoride (PMSF), pH 8.0]. The homogenate was centrifuged at 12,000 rpm for 20 min at 4°C. The supernatants were collected for Western blot analysis, which was performed as described previously (Kong et al., 2015). The antibodies used in the present study included anti-Mrp-2 (1:500; sc-5770, Santa Cruz; California, USA), anti-glyceraldehyde 3-phosphate dehydrogenase (anti-GAPDH) (1:20,000; YM3029, Immunoway; Texas, USA), anti-UGT1A1 (1:4,000; ab194697, Abcam; Cambs, UK), anti-P glycoprotein (1:1,000; ab170904, Abcam; Cambs, UK), anti-CES2 (1:1,000; DF6433, Affinity; Cambs, UK), and anti-Bcrp (1:200; sc-69988, Santa Cruz; California, USA). The gray intensities of the bands were quantified using ImageJ software (National Institute of Health, MD, USA).

### Statistical analysis

The pharmacokinetic parameters, including area under the concentration time curve to the respective sampling point (AUC_0−t_), area under the concentration time curve to infinity (AUC_0 → ∞_), initial plasma concentration (C_0_), maximum plasma concentration (C_max_), mean residence time (MRT), clearance rate (CL_obs_), volume of distribution (V_d_), and half-life (t_1/2_), were evaluated using Phoenix WinNonlin 6.0 (Certara, USA). Statistical comparisons were performed using Student's unpaired *t*-tests (SPSS 16.0 software). The data were expressed as the means ± standard error of the mean (SEM). The statistical significance was considered at *p* < 0.05.

## Results

### UHPLC-MS/MS method development

**Figure 1:**
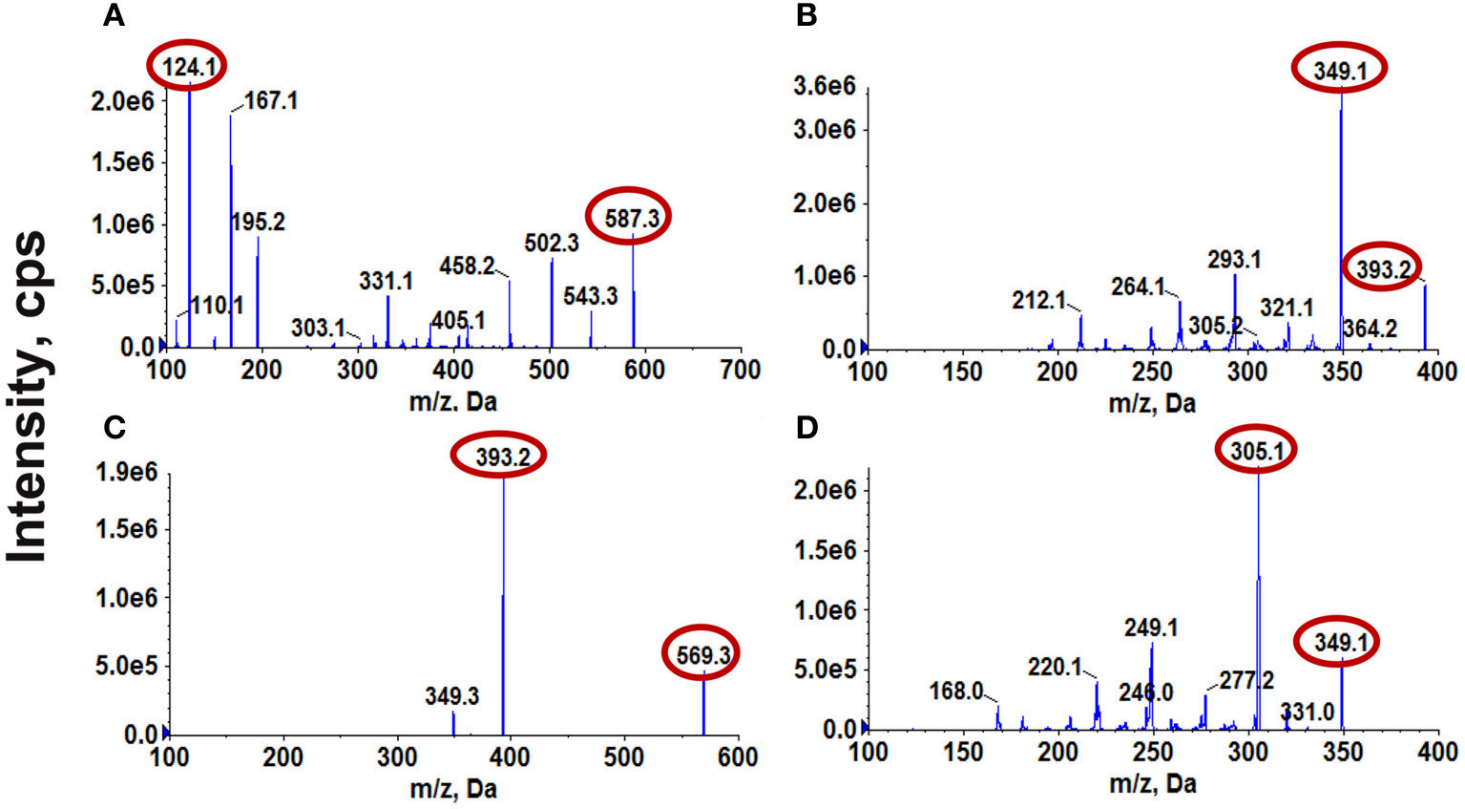
Mass spectra of CPT-11 **(A)**, SN-38 **(B)**, SN-38G **(C)**, and CPT (ISTD) **(D)**.

**Figure 2:**
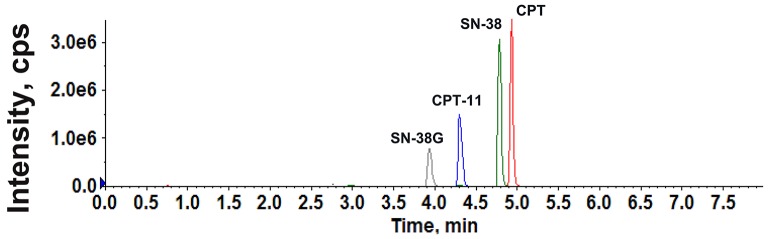
The total ion current chromatograms of CPT-11, SN-38, SN-38G, and CPT (ISTD).

ESI was performed in positive ionization mode for CPT-11, SN-38, and SN-38G. All of the analyzed components were quantified in multiple reaction monitoring (MRM) mode. CPT was selected as the internal standard (ISTD) because it showed a strong MS response in positive ion mode and exhibited chromatographic behavior similar to those of CPT-11, SN-38, and SN-38G. The precursor and product ion pairs for MRM detection and their corresponding declustering potential (DP), collision energy (CE), entrance potential (EP), and collision cell exit potential (CXP) values were optimized to obtain the maximum sensitivity and response. The results are presented in Figure 1 and Table 1. A gradient elution with 0.1% formic acid in water and acetonitrile was adopted for the LC to achieve satisfactory sensitivity and good peak shapes (Figure 2).

**Table 1:** MRM parameters of CPT-11, SN-38, SN-38G, and CPT (ISTD).

**Analytes**	**Precursor *m/z***	**Production *m/z***	**DP**	**CE**	**EP**	**CXP**
CPT-11	587.3	124.1	192	45	10	18
SN-38	393.2	349.1	197	34	9	17
SN-38G	569.3	393.2	226	37	10	19
CPT	349.1	305.1	210	32	9	13

### Method validation

The method was validated for its selectivity, calibration curve, sensitivity, precision, accuracy, recovery, matrix effect, dilution integrity, and stability (as shown in Supplementary Information), in compliance with the guidelines on bioanalytical method validation from the European Medicines Agency (European Medicine Agency, 2011) and the US Food and Drug Administration (FDA) [Center for Drug Evaluation and Research (CDER) and US Food and Drug Administration, 2001].The data obtained from method validation are shown in Figure S1 and Tables S1–S8. All of the results were acceptable, indicating that the method can reliably determine the CPT-11, SN-38, and SN-38G concentrations in the tested matrixes.

### Effect of SXD on the pharmacokinetics of CPT-11, SN-38, and SN-38G

**Figure 3:**
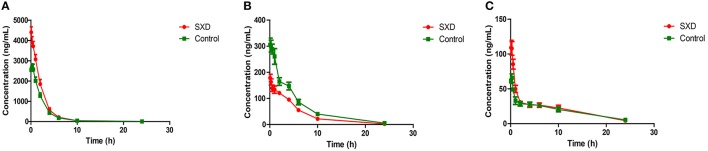
Mean plasma concentration-time profiles of CPT-11 **(A)**, SN-38 **(B)**, and SN-38G **(C)** following the i.v. administration of CPT-11 at 20 mg/kg in the control and SXD-pre-treated rats.

The developed method was successfully applied to a pharmacokinetics study of CPT-11 in SD rats after the i.v. administration of CPT-11. The mean plasma concentration-time profiles of CPT-11 and its metabolites (SN-38 and SN-38G) are shown in Figure 3. The primary pharmacokinetics parameters of CPT-11, SN-38, and SN-38G are listed in Table 2. In the SXD-pre-treated rats, the corresponding AUC and C_0_ of CPT-11 were significantly higher than those in the control group (*p* < 0.05 and *p* < 0.001, respectively), whereas the AUC and C_max_ of SN-38 were clearly reduced (*p* < 0.01 and *p* < 0.001, respectively). Moreover, the pretreatment of rats with SXD significantly decreased the elimination CL of CPT-11. The V_d_ of CPT-11 also decreased, although this difference did not reach statistical significance. SN-38G exhibited a significant (*p* < 0.01) increase in its C_max_ after pretreatment with SXD. However, no significant variations were found for MRT and *t*_(1/2)_ between the control group and the SXD-pre-treated group. The decreased AUC ratio of SN-38 to CPT-11 in the SXD group may suggest the inhibition of the metabolic transformation of CPT-11 to SN-38, while the significantly increased AUC ratio of SN-38G to SN-38 indicated that the glucuronidation of SN-38 was accelerated (Paoluzzi et al., 2004; Jong et al., 2007).

**Table 2:** Pharmacokinetic parameters of CPT-11, SN-38, and SN-38G in control and SXD-treated rats after intravenous administration of CPT-11 at 20 mg/kg (*n* = 6, Mean ± SEM).

**Analytes**	**Pharmacokinetic parameters**	**Groups**	
		**SXD-treated**	**Control**
CPT-11	AUC_0 → 24h_ (h^*^ng/mL)	10022.75 ± 1049.02^*^	7016.64 ± 661.96
	AUC_0 → ∞_ (h^*^ng/mL)	10070.14 ± 1056.34^*^	7100.89 ± 682.87
	C_0_ (ng/mL)	4690.05 ± 306.47^***^	2587.52 ± 148.94
	MRT (h)	1.90 ± 0.16	2.23 ± 0.16
	CL_obs_ (mL/h/kg)	2117.13 ± 258.05^*^	2947.11 ± 273.08
	V_d_ (mL/kg)	5747.81 ± 1051.19	10134.97 ± 1980.99
	*t*_(1/2)_ (h)	1.94 ± 0.37	2.42 ± 0.45
SN-38	AUC_0 → 24h_ (h^*^ng/mL)	963.02 ± 60.52^**^	1605.39 ± 165.09
	AUC_0 → ∞_ (h^*^ng/mL)	979.67 ± 67.41^**^	1642.37 ± 175.95
	C_max_ (ng/mL)	179.50 ± 34.31^***^	325.33 ± 26.45
	MRT (h)	4.77 ± 0.32	4.93 ± 0.23
	*t*_(1/2)_ (h)	3.89 ± 0.34	4.33 ± 0.29
	AUC ratio SN-38/CPT-11 (%)	10.18 ± 1.50^***^	22.84 ± 2.02
SN-38G	AUC_0 → 24h_ (h^*^ng/mL)	518.80 ± 38.19	420.89 ± 57.47
	AUC_0 → ∞_ (h^*^ng/mL)	590.43 ± 39.43	495.02 ± 89.53
	C_max_ (ng/mL)	117.53 ± 9.88^**^	63.03 ± 8.19
	MRT (h)	6.98 ± 0.43	7.51 ± 0.45
	*t*_(1/2)_ (h)	8.17 ± 2.36	7.66 ± 1.43
	AUC ratio SN-38G/SN-38 (%)	54.62 ± 4.29^**^	27.69 ± 4.98

*Statistical difference between control and SXD-treated rats*,

* *p < 0.05*,

** *p < 0.01*,

*** *p < 0.001*.

### Effect of SXD on the biliary excretions of CPT-11, SN-38, and SN-38G

**Figure 4:**
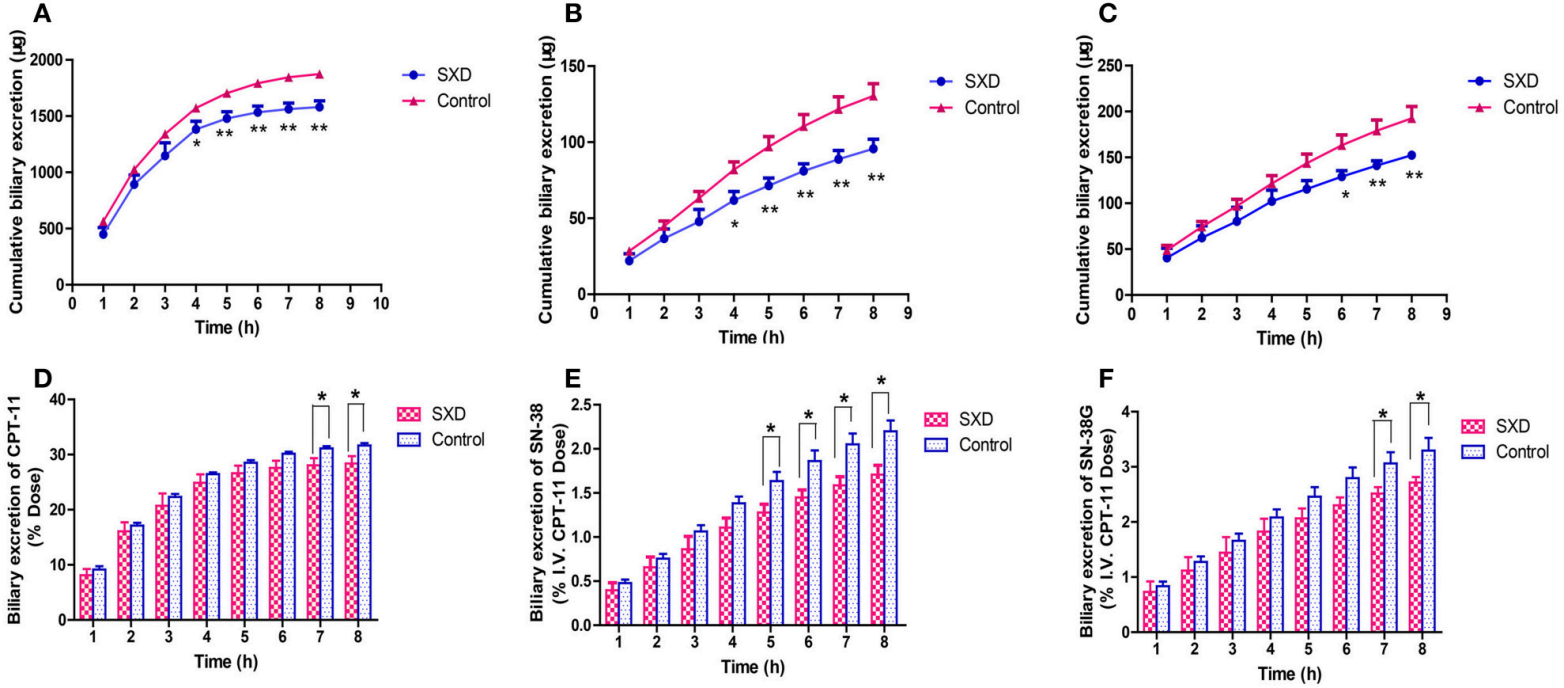
Cumulative biliary excretions of CPT-11 **(A)**, SN-38 **(B)**, and SN-38G **(C)** following the i.v. administration of CPT-11. Biliary excretions of CPT-11 **(D)**, SN-38 **(E)**, and SN-38G **(F)** over 8 h expressed as percentages of the CPT-11 dose. The data are presented as the mean ± SEM (*n* = 6). ^*^p < 0.05, ^**^p < 0.01, significantly different from the control.

Figure 4 shows the cumulative biliary excretions of CPT-11, SN-38, and SN-38G for 8 h following i.v. administration in control and SXD-pre-treated rats. In the SXD-pre-treated group, the cumulative biliary excretions of CPT-11 and SN-38 decreased significantly from 3 to 8 h, whereas that of SN-38G decreased from 5 to 8 h. The pretreatment of rats with SXD decreased the cumulative biliary excretion of CPT-11 over 8 h from 1873.77 ± 28.76 to 1580.39 ± 54.93 μg (*p* < 0.01). Similarly, the pretreatment decreased the cumulative biliary excretions of SN-38 and SN-38G significantly from 130.44 ± 7.99 to 95.67 ± 6.23 (*p* < 0.01) and from 192.54 ± 12.97 to 152.36 ± 4.67 μg (*p* < 0.01), respectively. The biliary excretions of CPT-11, SN-38, and SN-38G over 8 h, expressed as percentages of the CPT-11 dose, are shown in Figure 4. Following the i.v. administration of CPT-11, approximately 32% of the CPT-11 dose was excreted into bile over 8 h, whereas this value was reduced to 28% in the SXD-pre-treated group (*p* < 0.05). The biliary excretions of both SN-38 and SN-38G were also reduced by SXD pretreatment.

### Effect of SXD on the CPT-11, SN-38, and SN-38G concentrations in the intestinal contents

**Figure 5:**
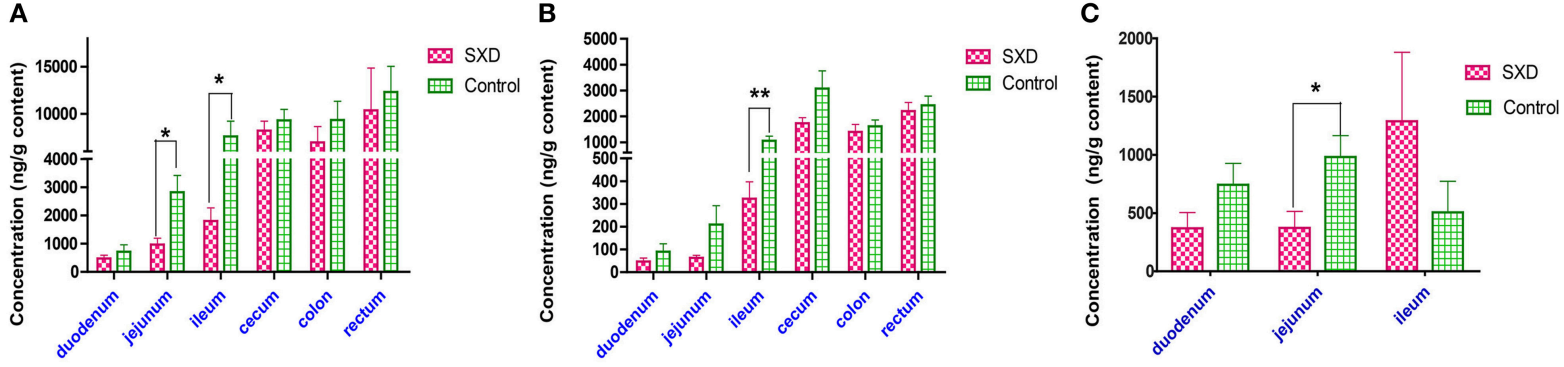
Effect of SXD on the concentrations of CPT-11 **(A)**, SN-38 **(B)**, and SN-38G **(C)** in the intestinal contents after the i.v. administration of CPT-11 in rats at 20 mg/kg. Each bar represents the mean ± SEM from six different rats. ^*^p < 0.05, ^**^p < 0.01, statistically significant difference between the control and SXD-pre-treated rats.

The CPT-11, SN-38, and SN-38G concentrations in the duodenal, jejunal, ileal, cecal, colonic, and rectal contents from the SXD-pre-treated and control groups are shown in Figure 5. The CPT-11 and SN-38 concentrations in the large intestinal (cecum, colon, and rectum) contents were higher than those in the contents from the small intestinal (duodenum, jejunum, and ileum) lumens. However, SN-38G was hardly detected in the large intestinal contents of the two groups. Compared with the control group, SXD pretreatment decreased the concentrations of CPT-11 and SN-38 in all of the intestinal contents to some extent. The CPT-11 concentrations in the jejunal and ileal contents of the SXD-pre-treated rats were only 34.21 and 23.76%, respectively, of those in the control group. The concentrations of SN-38 in the ileal contents and SN-38G in the jejunal contents were reduced to 30.55 and 37.92%, respectively. Notably, the SN-38G concentration in the ileal content increased.

### Effect of SXD on the CPT-11, SN-38, and SN-38G concentrations in the liver and intestinal tissues

**Figure 6:**
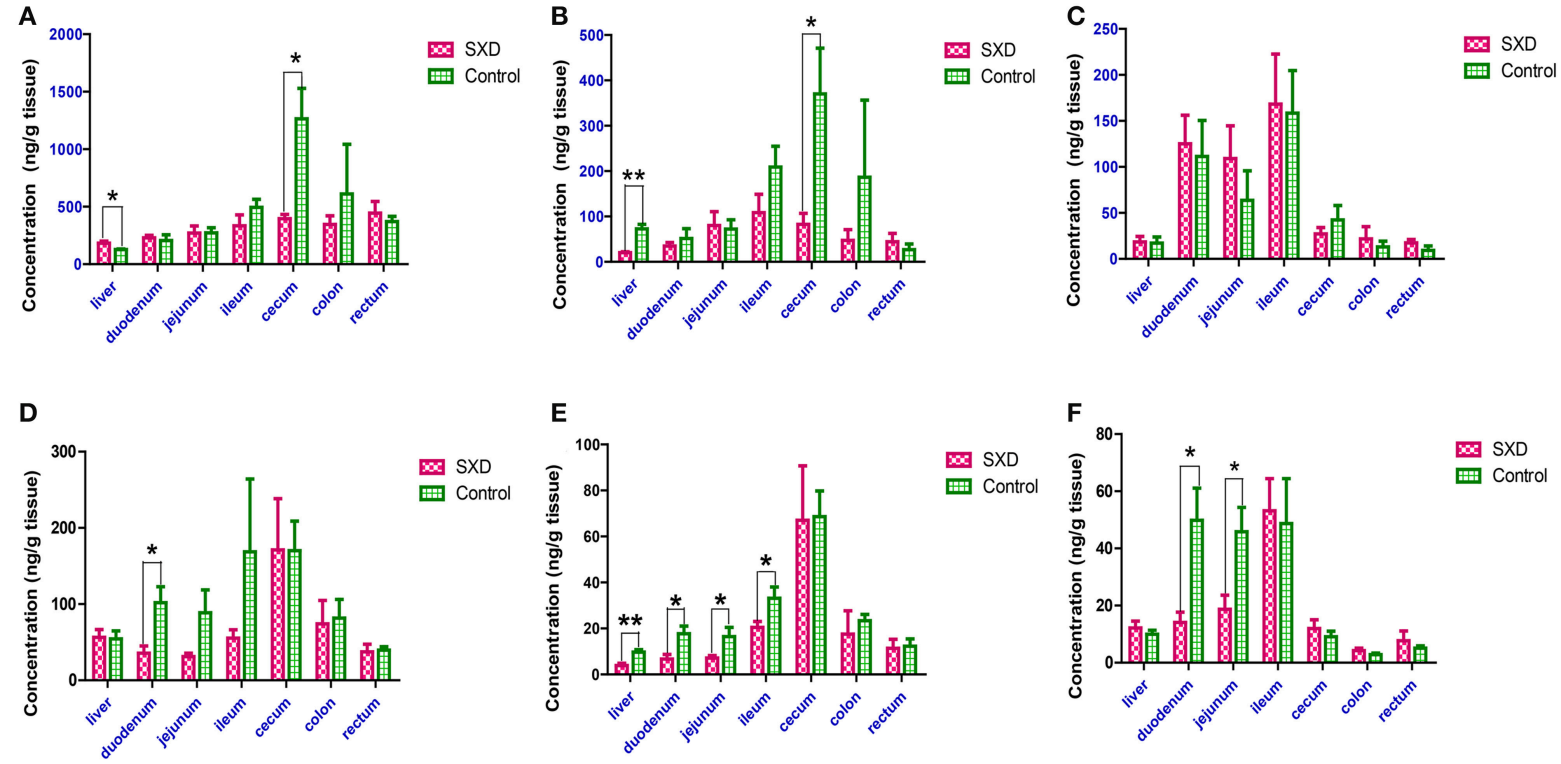
Effect of SXD on the tissue concentrations of CPT-11 **(A,D)**, SN-38 **(B,E)**, and SN-38G **(C**,**F)** at 8 h **(A–C)** and 24 h **(D–F)** after the i.v. administration of CPT-11 at 20 mg/kg. The data are the mean ± SEM of six independent determinations. ^*^p < 0.05, ^**^p < 0.01 vs. control.

Figure 6 shows the tissue levels of CPT-11 and its metabolites in the liver, duodenum, jejunum, ileum, cecum, colon, and rectum at 8 and 24 h after the i.v. administration of CPT-11 to the control and SXD-pre-treated rats. Regarding the concentrations of CPT-11 and its metabolites in the liver, the effects of SXD on the hepatic concentrations of SN-38G were insignificant, whereas SXD increased the hepatic CPT-11 concentration to 143.42% and reduced the SN-38 concentration to 27.66% relative to the values in the control group at 8 h after i.v. CPT-11 administration. Although, the SN-38 concentration in the liver remained lower than that in the control group (*p* < 0.01) at 24 h, no differences were found in the hepatic distributions of CPT-11 and SN-38G between the two groups.

In the control rats, the concentrations of CPT-11 and its metabolite SN-38 in the cecum tissue were higher than those in other intestinal segments. At 8 h after CPT-11 administration, SXD significantly decreased the concentrations of CPT-11 and SN-38 in the cecum to 31.26 and 22.36% (*p* < 0.05), respectively. Although, SXD reduced the concentrations of CPT-11 and SN-38 in the ileum and colon, these differences were not significant. The SN-38G concentration in the small intestinal tissue was slightly increased. At 24 h after i.v. administration, significant reductions in the concentrations of CPT-11, SN-38, and SN-38G were observed in the duodenum. Compared with those in the control rats, the SN-38 concentrations in the jejunum and ileum tissues and that of SN-38G in the jejunum from SXD-pre-treated rats were decreased to 43.14, 61.87, and 40.76 (*p* < 0.05), respectively. SXD also decreased the CPT-11 concentrations in the jejunum and ileum tissues but increased the tissue accumulation of SN-38G in the ileum; however, these differences were not significant.

### Effects of SXD on the hydrolysis of CPT-11 and glucuronidation of SN-38 by hepatic and jejunal S9 fractions

**Figure 7:**
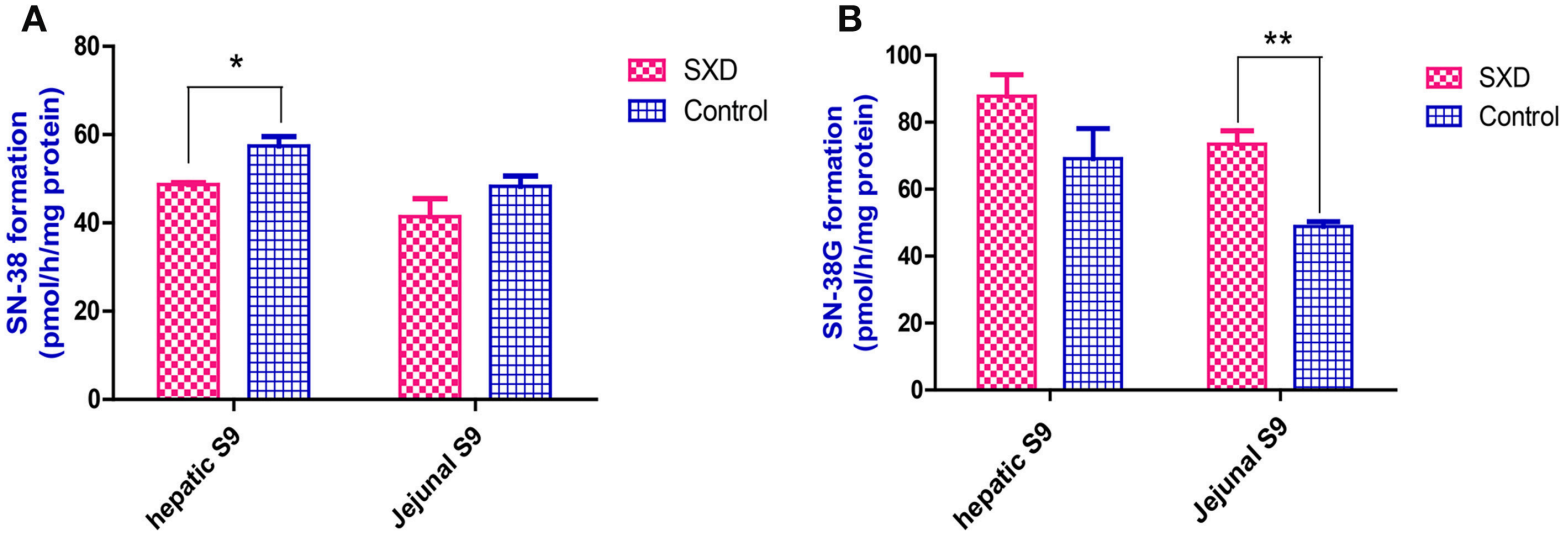
The hydrolysis of CPT-11 **(A)** and glucuronidation of SN-38 **(B)** in both the hepatic and jejunal S9 fractions. The hydrolysis and glucuronidation rates were calculated in pmol/h/mg protein. The data are shown as the mean ± SEM of at least three independent determinations and were analyzed by Student's *t*-test. The asterisk (^*^) indicates a significant difference compared with the control, ^*^p < 0.05, ^**^p < 0.01.

The production of SN-38 from CPT-11 over a 2-h incubation period with the hepatic and jejunal S9 fractions from control and SXD-pre-treated rats is shown in Figure 7. The SN-38 level in the hepatic S9 fraction of SXD-pre-treated rats was only 84.81% of that of the control rats (*p* < 0.05), which suggested that the CES-mediated hydrolysis of CPT-11 was lower in the liver S9 fractions from SXD-pre-treated rats. This finding implied that the conversion from CPT-11 to SN-38 in the liver was hindered by SXD. Similarly, the higher SN-38G concentration produced from SN-38 in the jejunal S9 fraction of SXD-pre-treated rats indicated that the SXD pre-treated rats had a greater SN-38 glucuronidation capacity than the control rats. Differences were observed in the CPT-11 hydrolysis in the jejunal S9 fraction and SN-38 glucuronidation in the hepatic S9 fraction between the two groups, although these differences were not significant.

### Effect of SXD on CES2, UGT1A1, Mrp-2, Bcrp, and P-gp expression

**Figure 8:**
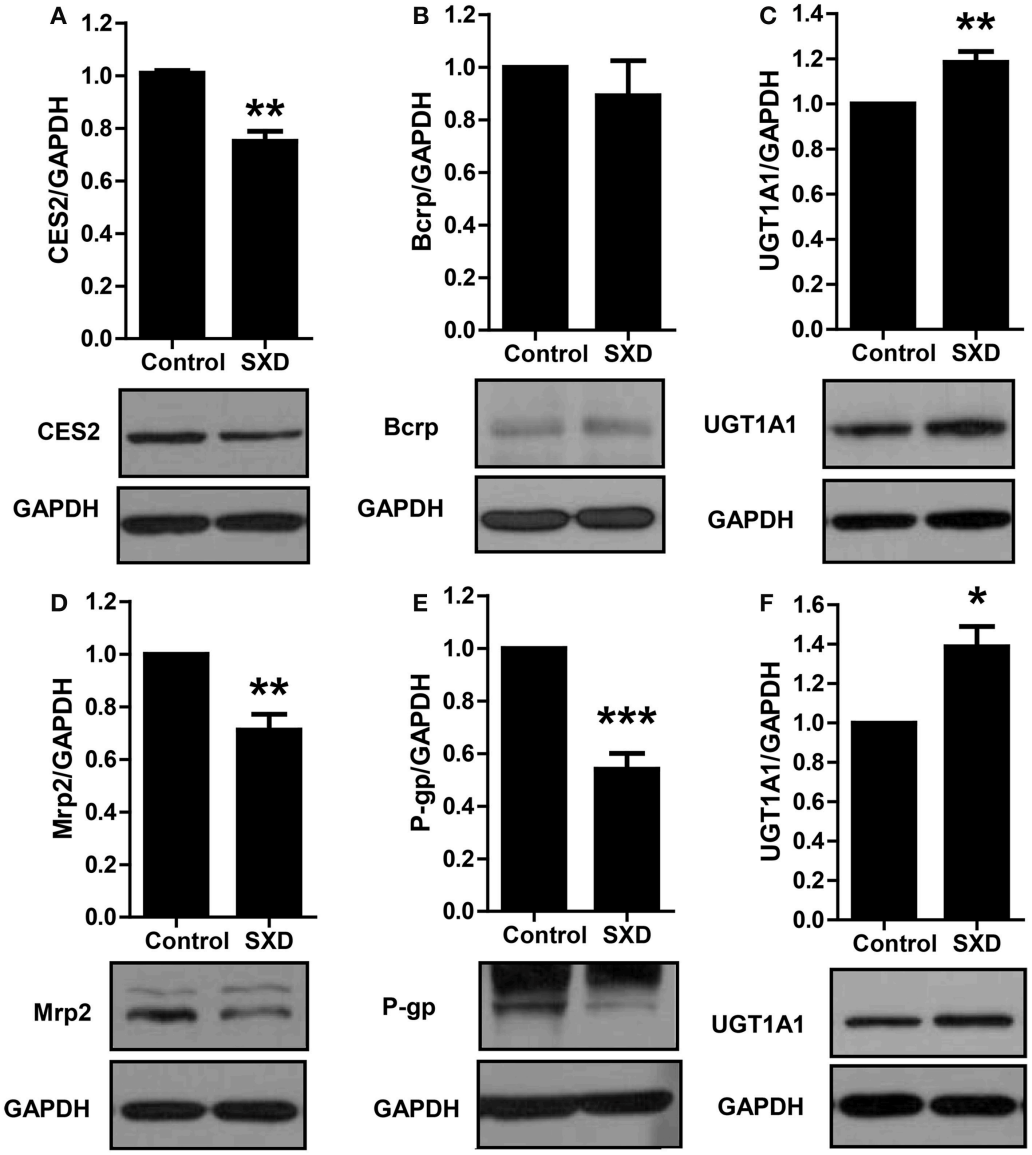
Effect of SXD on the hepatic expressions of CES2 **(A)**, Bcrp **(B)**, UGT1A1 **(C)**, Mrp-2 **(D)**, P-gp **(E)**, and the jejunal expression of UGT1A1 **(F)**. Data are expressed as mean ± SEM (*n* = 9). ^*^Statistically significant differences are shown (^*^p < 0.05, ^**^p < 0.01, and ^***^p < 0.001) relative to the control.

Potential changes in the expression of CES2, UGT1A1, Mrp-2, Bcrp, and P-gp in rat liver and UGT1A1 in jejunal segment from the control and SXD-pre-treated groups were explored by Western blot analyses (Figure 8, Figures S2–S7). The expression of Mrp-2, P-gp, and CES2 in the liver was significantly decreased in the SXD-pre-treated rats (*p* < 0.01, *p* < 0.001), whereas the expression of UGT1A1 in the liver and jejunum was increased (*p* < 0.05, *p* < 0.01). No significant difference in Bcrp protein expression was observed.

## Discussion

In this study, a sensitive, accurate and rapid UHPLC-ESI-MS/MS method was developed and validated for the simultaneous determination of CPT-11 and its metabolites (SN-38 and SN-38G) in rat plasma, bile, liver, and intestine and intestinal contents. The sample preparation method almost completely eliminated the interference caused by the presence of different biological matrixes. The developed method was successfully used for a pharmacokinetic and tissue distribution study of CPT-11 and its metabolites.

The pharmacokinetic results revealed that SXD increased the CPT-11 plasma concentration and AUC and resulted in a concomitant decrease in its plasma clearance (CL). By contrast, the AUC and C_max_ of SN-38 were reduced. The alteration of the pharmacokinetics of CPT-11 by SXD differed from that by probenecid (Horikawa et al., 2002a) as an Mrp-2 inhibitor and that by verapamil (Bansal et al., 2009) as a P-gp inhibitor, which increased the plasma levels of both CPT-11 and SN-38. This result suggested that SXD might change the expression or activity of metabolic enzymes. The significantly decreased SN-38/CPT-11 AUC ratio in the SXD-pre-treated group implied that the conversion of CPT-11 to SN-38 was hindered by SXD. The decreased hepatic expression of CES2, which is a key enzyme involved in the conversion of CPT-11 to SN-38 (Humerickhouse et al., 2000; Sanghani et al., 2003), provided direct evidence supporting the abovementioned speculation. In addition, the decreased hydrolysis of CPT-11 to SN-38 in the liver S9 fraction from the SXD-pre-treated group further confirmed that the hydrolysis of CPT-11 to SN-38 was inhibited by SXD. Although, the initial plasma level of SN-38 decreased, the C_max_ of SN-38G from SN-38 glucuronidation increased in the SXD-pre-treated group. Moreover, the SN-38G/SN-38 AUC ratio was significantly higher than that in the control group. These observations implied that the glucuronidation of SN-38 to SN-38G might be strengthened by SXD. This presumption was confirmed by the up-regulation of UGT1A1 expression detected by Western blot analysis and the accelerated glucuronidation of SN-38 by hepatic and jejunal S9 fractions of SXD pre-treated rats.

Since SXD may exert its effects *via* multiple pathways, monitoring the plasma concentration alone was not sufficient for evaluating the effect of SXD on the pharmacokinetics of CPT-11. Hence, biliary excretion studies were also performed. The biliary excretions of CPT-11 and its metabolites were reportedly mediated by P-gp, Mrp-2, and Bcrp expressed at the bile canalicular membrane (Smith et al., 2006; Yokoo et al., 2007). Similarly to probenecid (Horikawa et al., 2002a) or verapamil (Bansal et al., 2009), SXD decreased the biliary excretion of CPT-11 (mediated by P-gp and Mrp-2) with respect to its increased hepatic concentration (partly contributed by the inhibited conversion of CPT-11), which implied that SXD might regulate one or more hepatic efflux transporters. Considering the decreased concentration of SN-38 in the liver, the observed decreased biliary excretion of SN-38 might be due to the inhibited production of SN-38 from CPT-11 and/or the inhibited biliary clearance of SN-38 mainly mediated by Mrp-2 (Horikawa et al., 2002a) and Bcrp (Yokoo et al., 2007) by SXD. Compared with the alteration of SN-38G by probenecid, the SN-38G biliary excretion was also decreased, whereas the hepatic concentration was unchanged, suggesting that SXD might to some extent alter the biliary clearance of SN-38G in addition to metabolic enzymes.

The reduced cumulative biliary excretions of CPT-11, SN-38, and SN-38G by SXD directly resulted in decreased CPT-11 and SN-38 concentrations in the small intestinal contents and decreased SN-38G concentrations in the duodenal and jejunal contents from the SXD-pre-treated group. The decreased concentrations of CPT-11 and SN-38 in the large intestinal contents, which exhibited large deviations, were related to the water contents and the volumes of the intestinal contents in the intestinal lumen. Since the large intestinal lumen in the rats that received SXD pretreatment was apparently larger than that in untreated rats, the concentrations of CPT-11 and SN-38 were diluted by the greater large intestinal content. The barely detected SN-38G level in the large intestinal contents could be attributed to the hydrolysis of SN-38G by bacterial β-glucuronidase (Slatter et al., 2000; Basu et al., 2016). The SN-38G concentration in the ileal content was increased, and this phenomenon could be attributed to the SXD-induced decrease in the bacterial β-glucuronidase activity in the distal ileum lumen (Deng et al., 2017).

P-gp and Mrp-2 were also expressed in the apical domains of various organs, such as the hepatocytes and enterocytes of the small intestine (Arimori et al., 2003). As previously mentioned, the increased tissue levels of CPT-11 in the liver at 8 h after CPT-11 administration may be attributed to the reduced bile excretions of CPT-11 mediated by P-gp and Mrp-2, and the inhibited conversion of CPT-11 to SN-38. The decreased hepatic exposure to SN-38, which was primarily caused by the inhibition of SN-38 conversion from CPT-11 by SXD, exhibited a paralleled change with that of the plasma level. The reduced concentrations of CPT-11 and SN-38 in most of the small intestinal segments from SXD-pre-treated rats at 24 h after CPT-11 administration can be ascribed to the reduced biliary cumulative excretions and decreased concentrations of CPT-11 and SN-38 in the intestinal contents, which directly affected the tissue uptake. The slightly increased concentrations of SN-38G in the small intestinal tissue at 8 h were due to the acceleration of SN-38 glucuronidation *via* UGT1A1 induction by SXD. However, the reduced concentrations of SN-38G in the duodenum and jejunum at 24 h might simply be due to the decreased concentration of its precursor (SN-38) in these tissues. In addition, the significantly decreased cecal concentrations of CPT-11 and SN-38 at 8 h may be associated with the intestinal bacteria-involving biotransformation of CPT-11 and SN-38 and the tissue blood flow.

**Figure 9:**
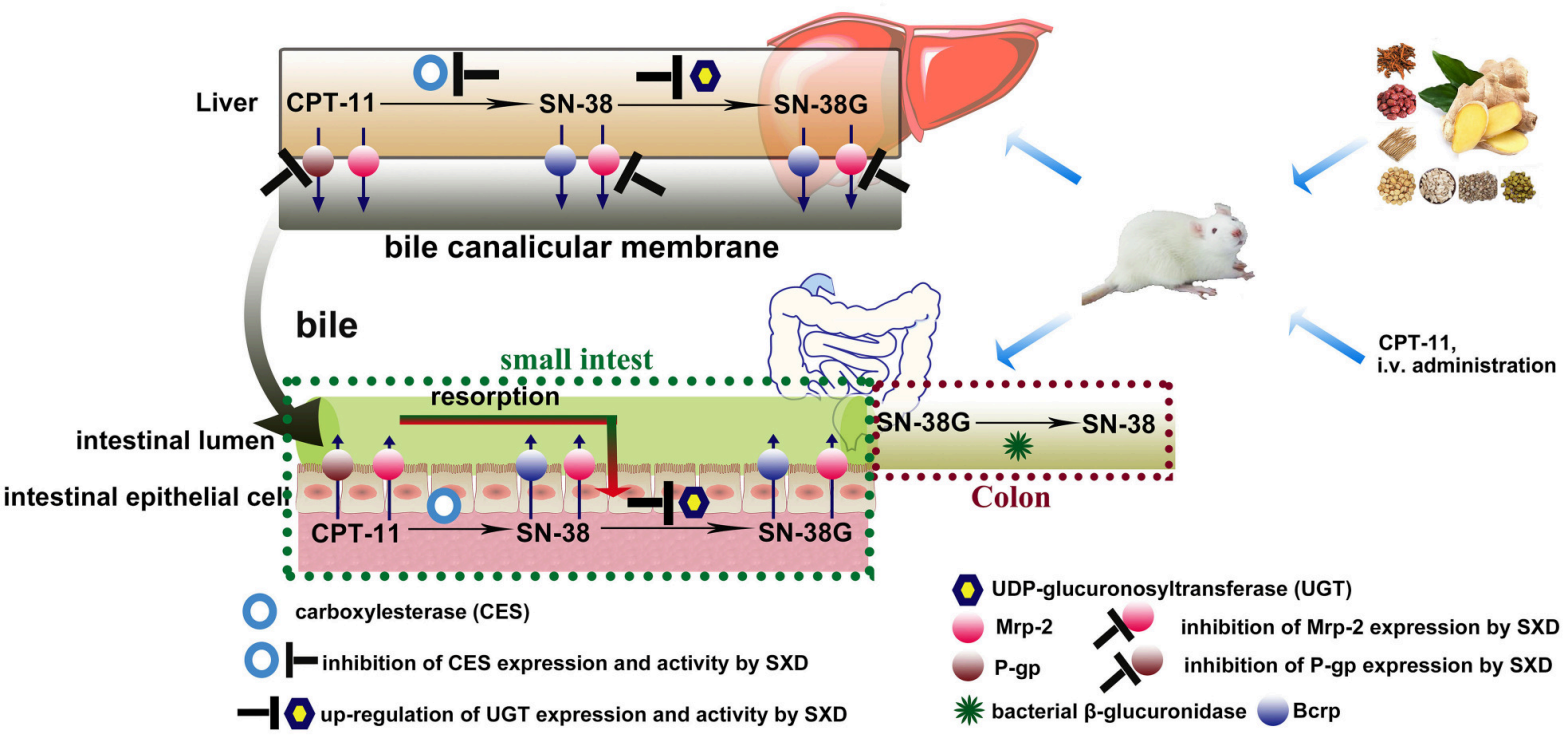
Key metabolic enzymes and transporters interacting with CPT-11 and its metabolites in the liver and intestinal epithelium. SXD altered the pharmacokinetics of CPT-11 by inhibiting Mrp-2 and P-gp expressions and altering the activities/expressions of CES2 and UDP-glucuronosyltransferase.

Diarrhea has been determined to be relevant to SN-38, and episodes of diarrhea have been found to be positively correlated with the AUC of SN-38 (Sasaki et al., 1995). Moreover, the gastrointestinal toxicity has been suggested to be caused by high levels of SN-38 and/or CPT-11 retained in the intestine (Araki et al., 1993). Based on the results of our study, SXD could alter the pharmacokinetics of CPT-11 and alleviate the diarrhea induced by CPT-11 by acting on multiple targets and pathways simultaneously (Figure 9). By decreasing the expression and activity of CES2, SXD inhibited the conversion of CPT-11 to SN-38 and eventually led to a decrease in the AUC of SN-38. Moreover, by reducing the Mrp-2- and P-gp-mediated cumulative elimination of CPT-11, SN-38, and SN-38G from the bile, SXD decreased the exposure of CPT-11 and its metabolites to the intestine. The cecum was reported to be most severely damaged by CPT-11 (Takasuna et al., 1996). Among the intestinal segments studied, the significant reduction in the cecal CPT-11 and SN-38 levels suggested that SXD mitigated the damage to the cecum induced by CPT-11 and SN-38. In addition, SXD decreased the SN-38 production by up-regulating the expression of UGT and inducing its glucuronidation. Notably, the intratumoral CPT-11 concentration and activation were essential not only to the toxicity of CPT-11 but also its antitumor activity (Kojima et al., 1998; Senter et al., 2001). The effect of SXD on the antitumor activity of CPT-11 should be further studied to rationalize the combined use of CPT-11 with SXD.

Glycyrrhizin and glycyrrhetinic acid have been reported to be inhibitors of Mrp-2 (Feng et al., 2015). The flavonoid oroxylin A is known to effectively inhibit P-gp-mediated drug efflux (Go et al., 2009). Moreover, chrysin has been found to induce UGT1A1 in the hepatoma cell line Hep G2 (Walle et al., 2000). As a TCM, SXD consists of the compounds described above and other active constituents. These active constituents exert their actions *via* different pathways and synergistically alter the pharmacokinetics of CPT-11. However, the inhibitors of CES enzymes present in SXD have not been extensively reported. Therefore, the inhibitors of CES enzymes and other active compounds targeting UGT1A1, P-gp, and Mrp-2 will be screened in our future study on SXD.

## Conclusion

A sensitive and accurate UHPLC-MS/MS method was developed and validated for the simultaneous quantification of CPT-11, SN-38, and SN-38G in rat plasma, bile, liver and intestine and intestinal contents after the i.v. administration of CPT-11. Using the developed method, the pharmacokinetic drug interaction between CPT-11 and SXD was shown. SXD could alter the pharmacokinetics of CPT-11 to alleviate CPT-11-induced diarrhea. The alterations were associated with multiple metabolic enzymes and transporters. The validation experiments proved that SXD could inhibit the hydrolysis of CPT-11 to SN-38 by CES and improve glucuronidation of SN-38 to SN-38G by UDP-glucuronosyltransferase. The Western blot analyses showed SXD down-regulated the hepatic expressions of CES2, Mrp-2, and P-gp, and up-regulated the hepatic and jejunal expressions of UGT1A1. The present study not only supplies pharmacokinetics data to explain the findings that treatment with SXD reduced the diarrhea induced by CPT-11, but also illustrates the underlying mechanism of the effects of SXD on the pharmacokinetics of CPT-11.

## Author contributions

YS and HG participated in research design. HG, PL, XW, JY, YH, XL, and MS were responsible for conducting experiments. YS and HG performed data analysis. YS, HG, and SL contributed to the writing and modification of the manuscript.

### Conflict of interest statement

The authors declare that the research was conducted in the absence of any commercial or financial relationships that could be construed as a potential conflict of interest.

## Supplementary material

Figure S1 Typical MRM chromatograms of **(a)** blank plasma; **(b)** blank plasma spiked with CPT-11, SN-38 and SN-38G at the LLOQ and CPT (ISTD); and **(c)** a plasma sample at 2 h following the i.v. administration of CPT-11.

Figure S2 The original western blot of CES2 in liver.

Figure S3 The original western blot of UGT1A1 in liver.

Figure S4 The original western blot of Mrp-2 in liver.

Figure S5 The original western blot of Bcrp in liver.

Figure S6 The original western blot of P-gp in liver.

Figure S7 The original western blot of UGT1A1 in jejunum.
