# Supplementary figures and images for: Shengjiang Xiexin Decoction Alters Pharmacokinetics of Irinotecan by Regulating Metabolic Enzymes and Transporters: A Multi-Target Therapy for Alleviating the Gastrointestinal Toxicity
Source: Front Pharmacol. 2017 Oct 27;8:769. doi: 10.3389/fphar.2017.00769 (PMC5663900; doi:10.3389/fphar.2017.00769)

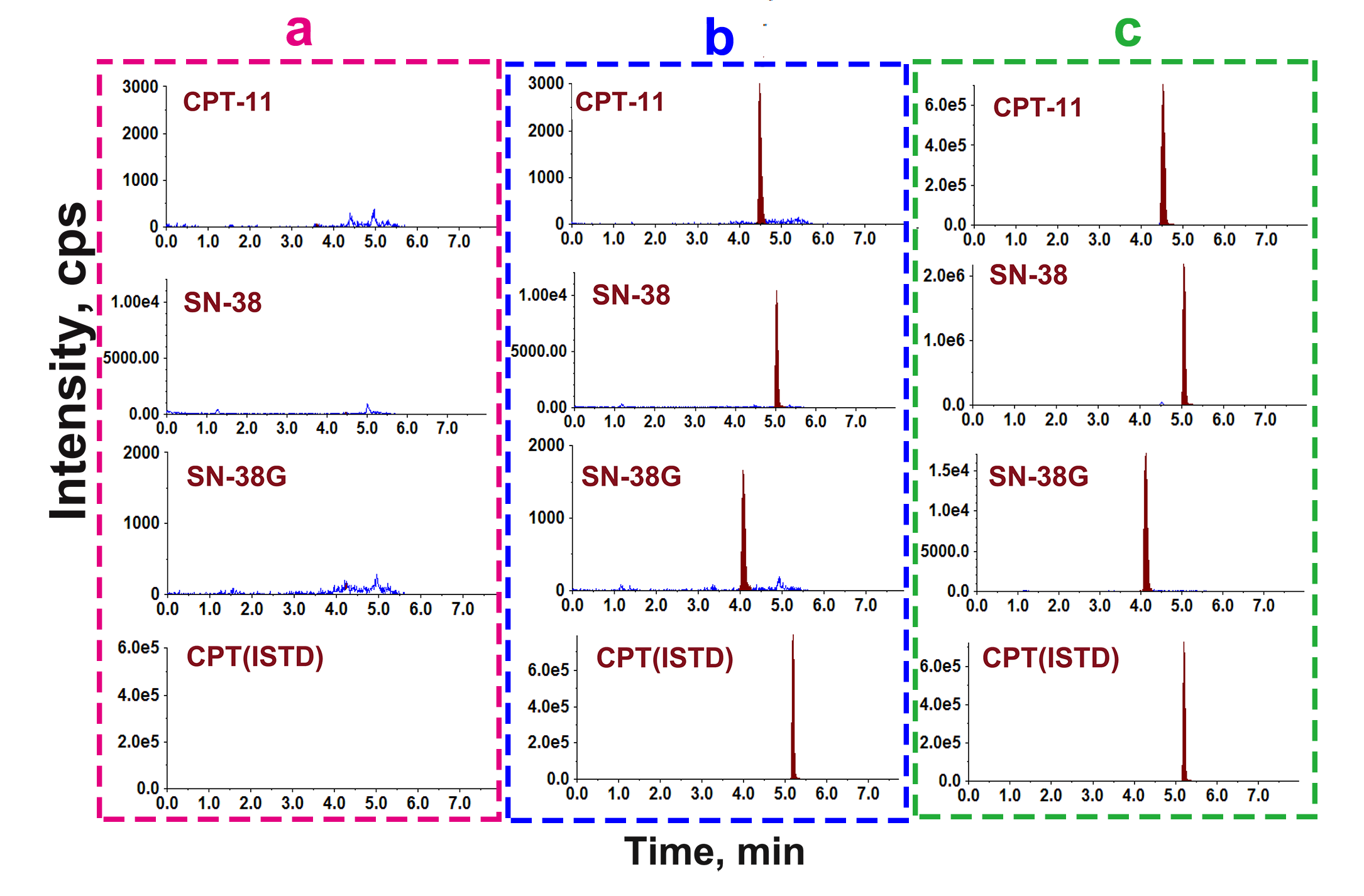

Supplement: Figure S1 — Typical MRM chromatograms of (a) blank plasma; (b) blank plasma spiked with CPT-11, SN-38 and SN-38G at the LLOQ and CPT (ISTD); and (c) a plasma sample at 2 h following the i.v. administration of CPT-11. [file Image1.TIFF]

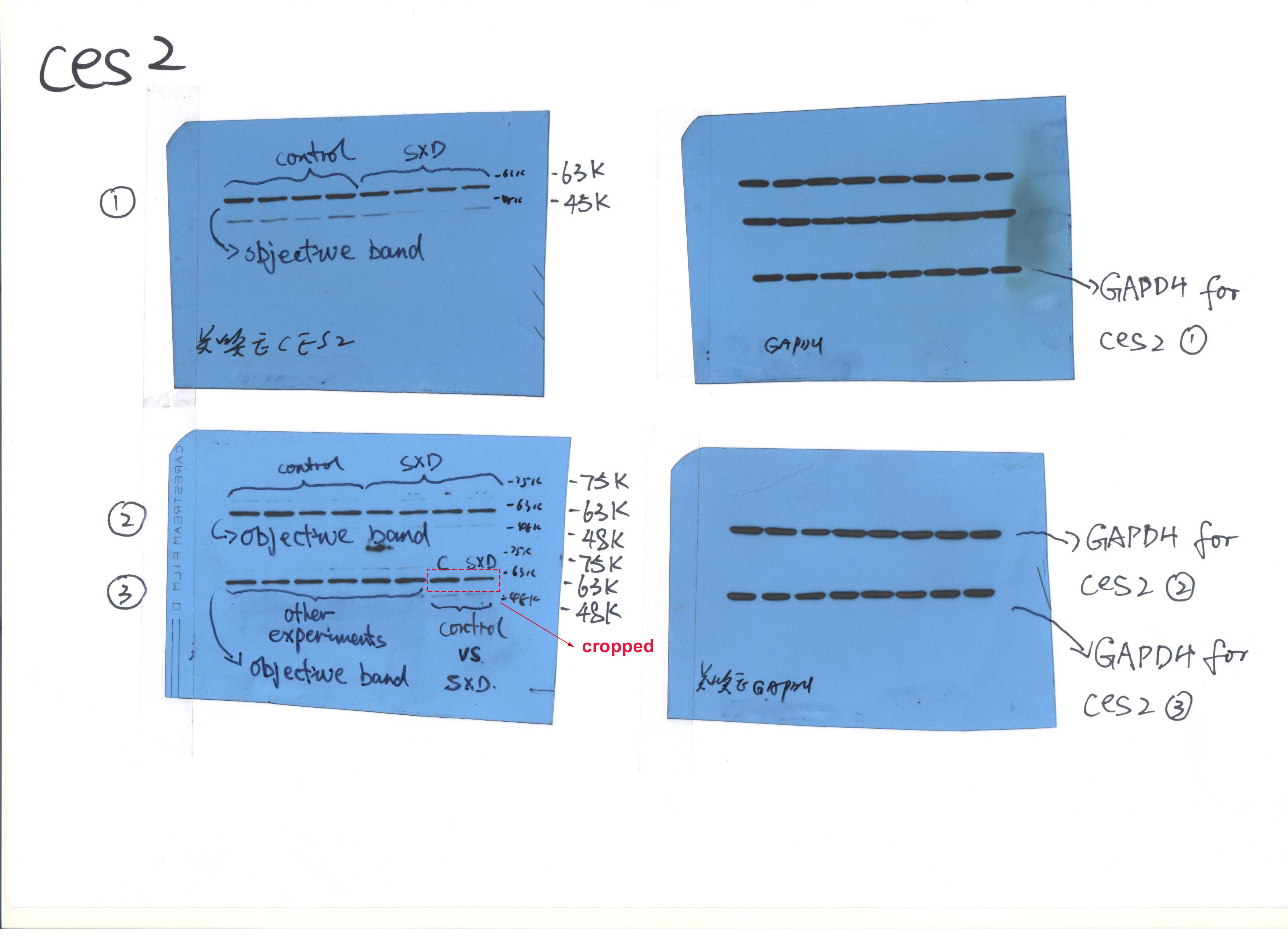

Supplement: Figure S2 — The original western blot of CES2 in liver. [file Image2.jpg]

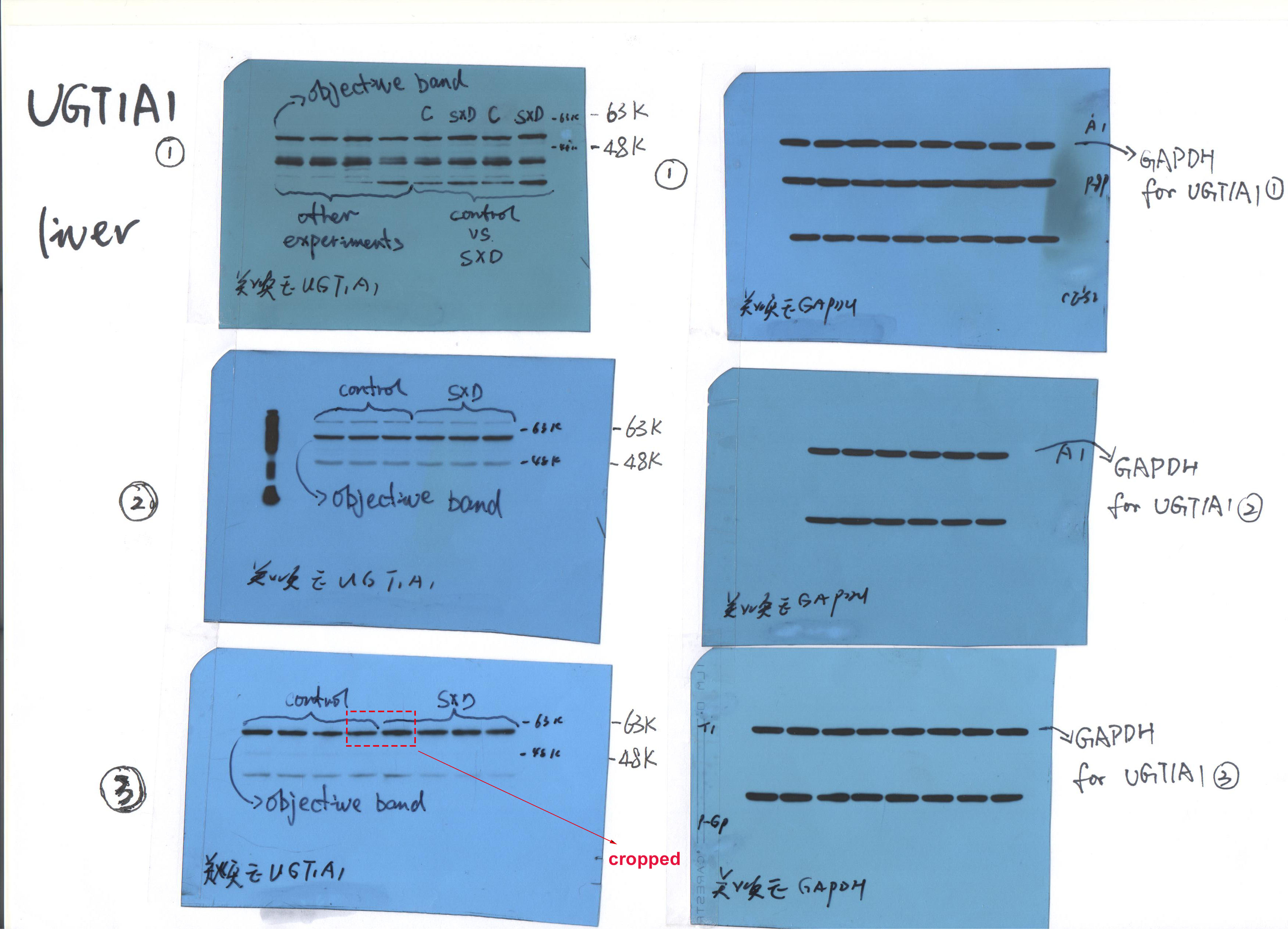

Supplement: Figure S3 — The original western blot of UGT1A1 in liver. [file Image3.jpg]

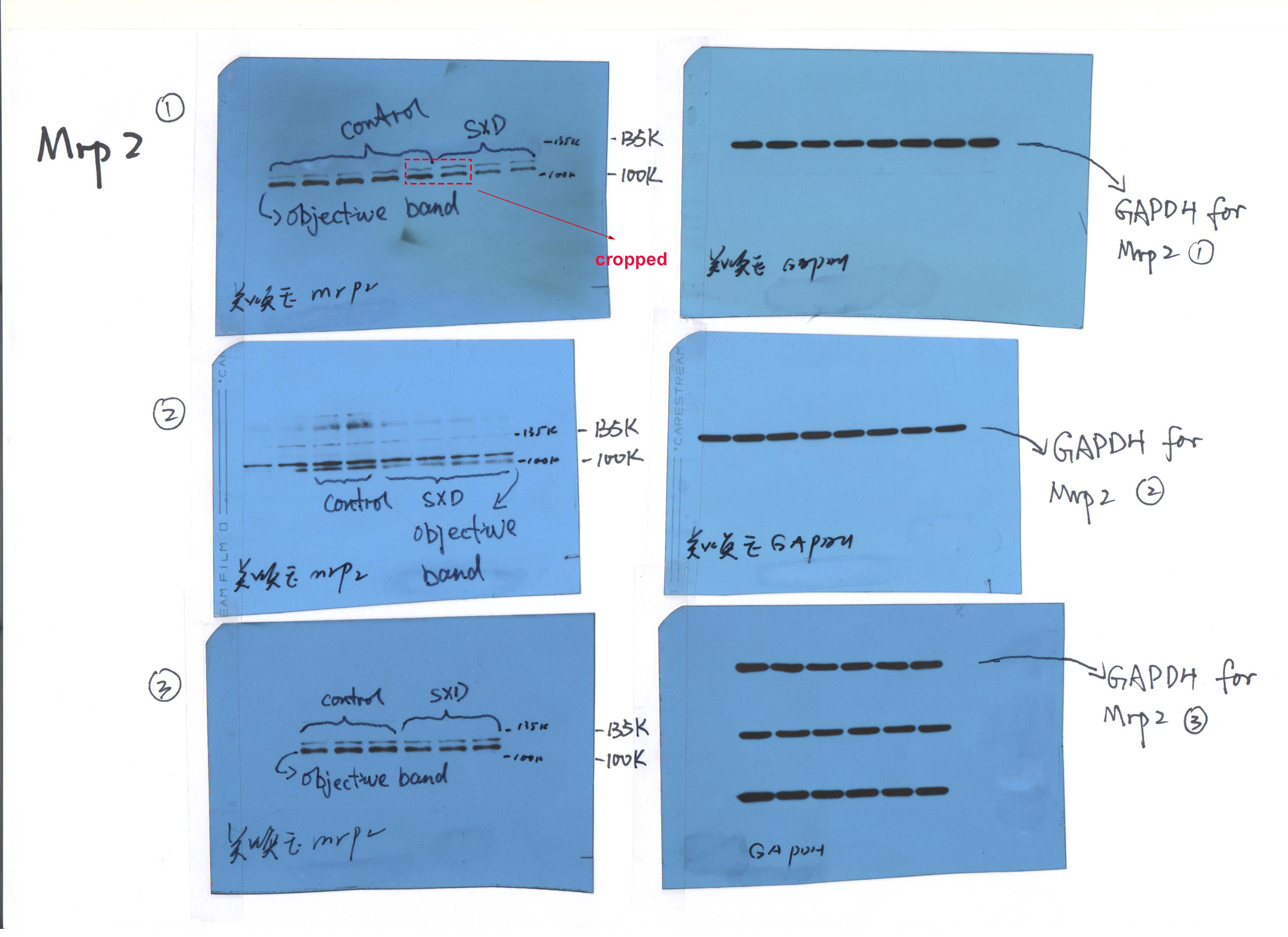

Supplement: Figure S4 — The original western blot of Mrp-2 in liver. [file Image4.jpg]

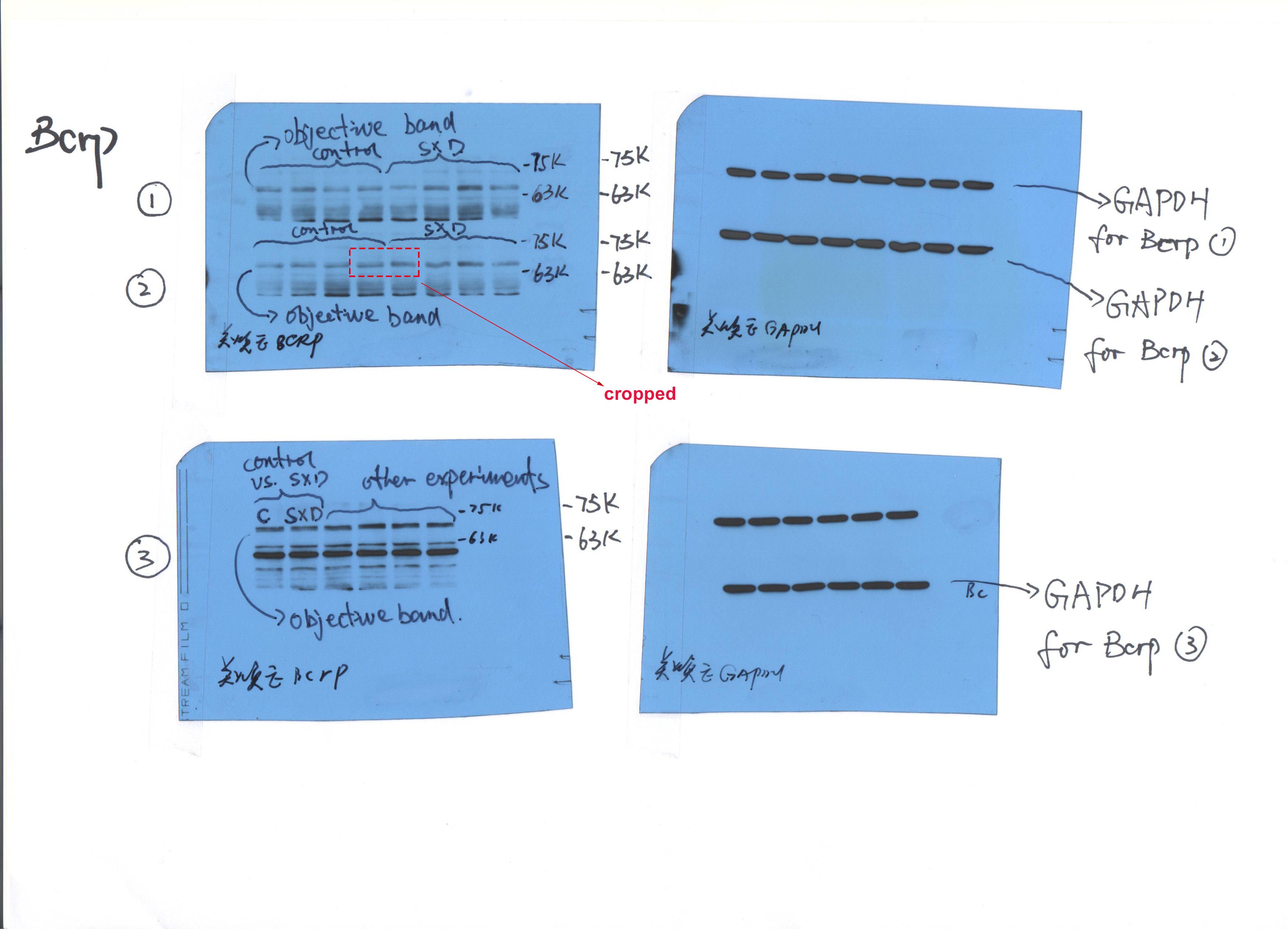

Supplement: Figure S5 — The original western blot of Bcrp in liver. [file Image5.jpg]

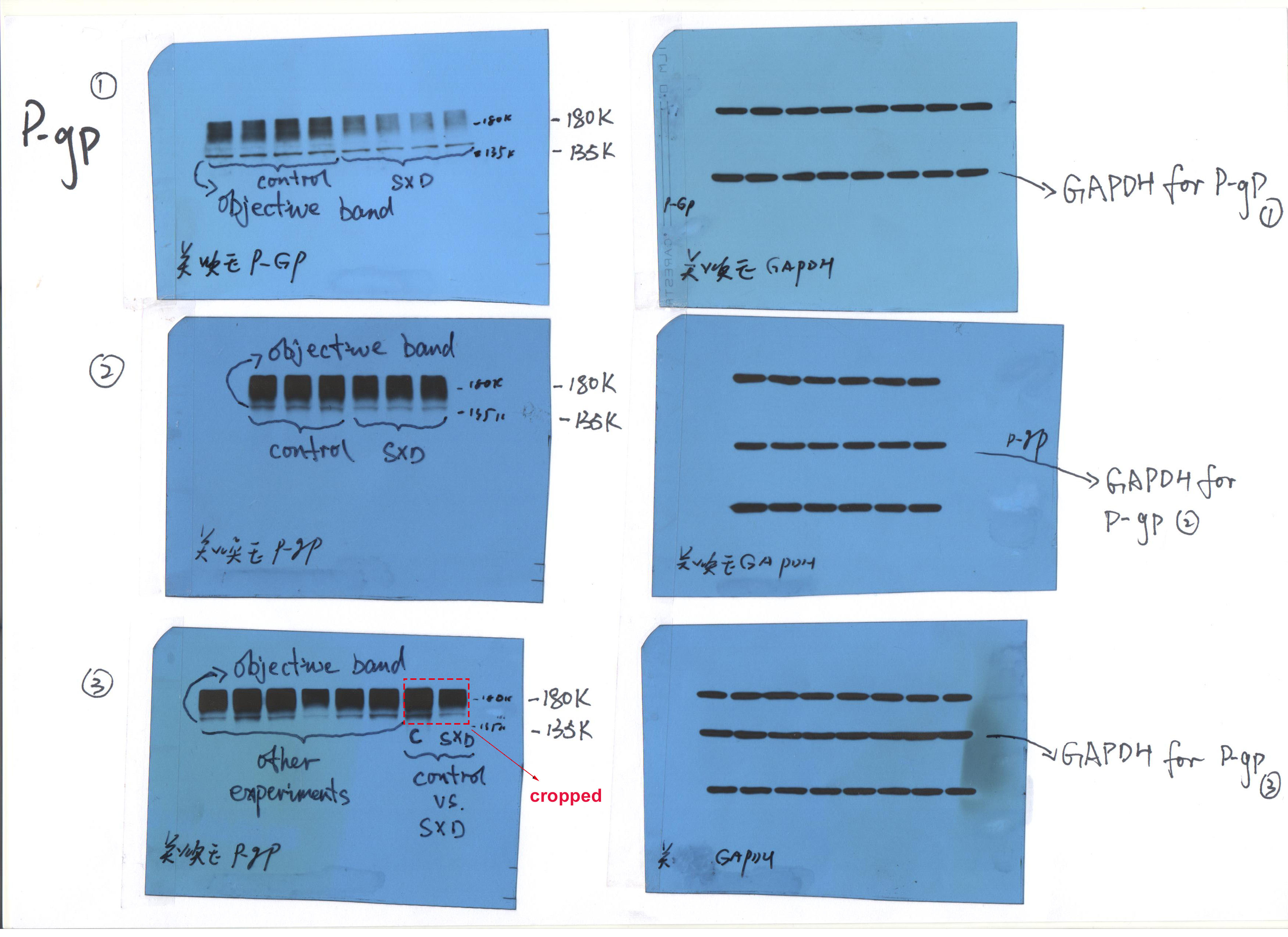

Supplement: Figure S6 — The original western blot of P-gp in liver. [file Image6.jpg]

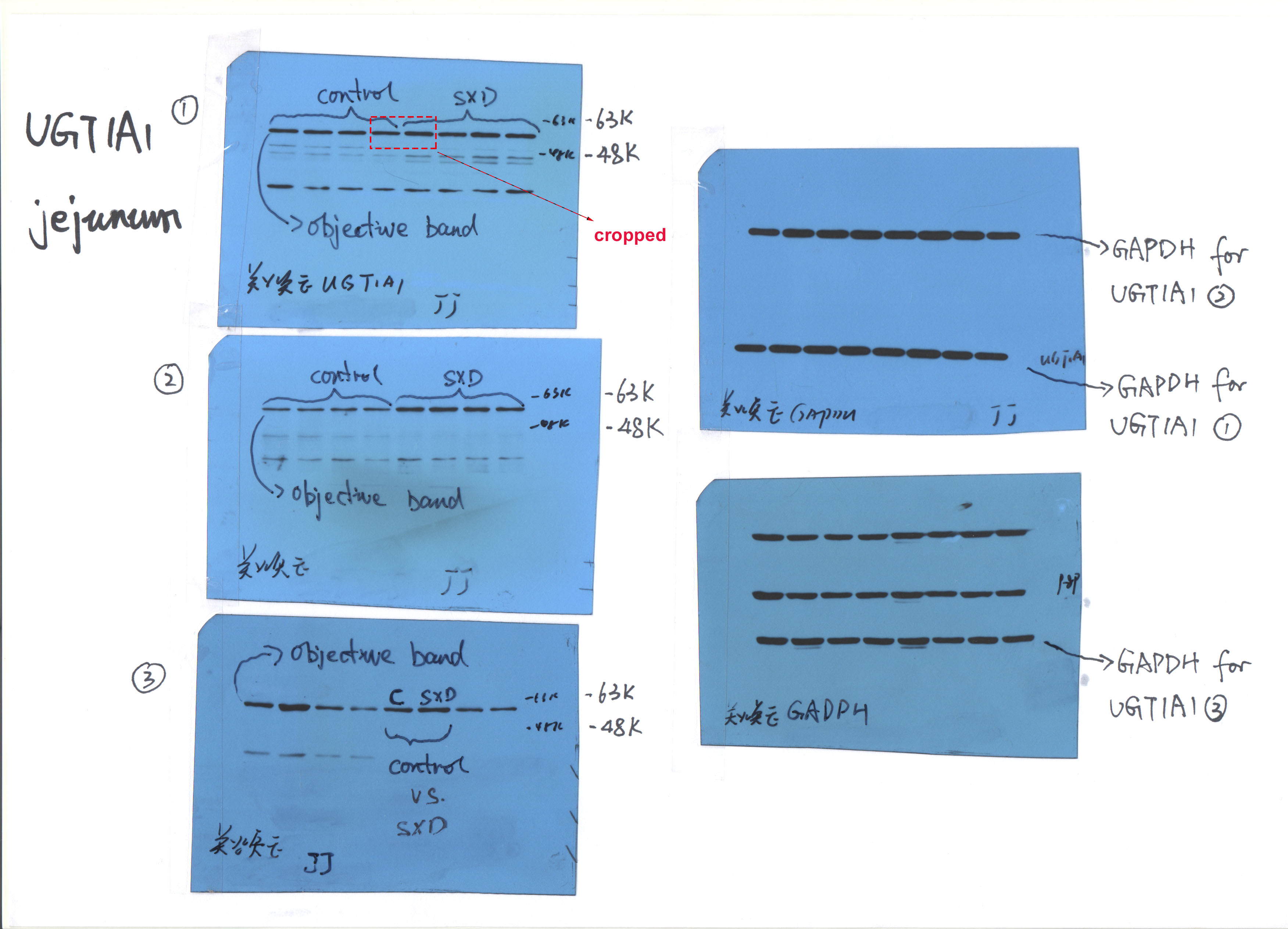

Supplement: Figure S7 — The original western blot of UGT1A1 in jejunum. [file Image7.jpg]
