# Supplementary material for: Shengjiang Xiexin Decoction Alters Pharmacokinetics of Irinotecan by Regulating Metabolic Enzymes and Transporters: A Multi-Target Therapy for Alleviating the Gastrointestinal Toxicity
Source: Front Pharmacol. 2017 Oct 27;8:769. doi: 10.3389/fphar.2017.00769 (PMC5663900; doi:10.3389/fphar.2017.00769)
Supplement: Supplementary file 8 [file Presentation1.pdf]

Supplementary Information

Shengjiang Xiexin Decoction Alters Pharmacokinetics of Irinotecan by Regulating Metabolic Enzymes and Transporters: A Multi-Target Therapy for Alleviating the Gastrointestinal Toxicity

Huan-yu Guan[1,2], Peng-fei Li[1], Xiao-ming Wang[1], Jia-jing Yue[1], Yang He[1], Xiao-mei Luo[1], Mei-feng Su[1], Shang-gao Liao[1] & Yue Shi[1 *]

[1] Institute of Medicinal Plant Development, Chinese Academy of Medical Sciences and Peking Union Medical College, Beijing, China.

[2] School of Pharmaceutical Sciences, Guizhou Medical University, Guiyang, China.

*** Correspondence:** Yue Shi: email: shiyue1029@126.com

Data from Method Validation

Selectivity

The selectivity of the method was evaluated by analyzing six individual batches of blank samples (plasma, bile, liver, intestine tissue and intestinal contents). The chromatographic findings for each blank sample prepared using the proposed pretreatment were compared to those of the corresponding spiked sample with the analytes and ISTD and relative to those of the samples obtained after the administration of CPT-11. Typical MRM chromatograms of a blank rat plasma sample; blank rat plasma samples spiked with CPT-11 (1.0 ng/mL), SN-38 (0.2 ng/mL), SN-38G (1.0 ng/mL) and ISTD (50 ng/mL); and a rat plasma sample collected 2 h after the i.v. administration of 20 mg/kg CPT-11 are shown in **Fig. S1**.

Calibration curve and LLOQ

The calibration standards and quality control (QC) samples of plasma, bile, liver, intestine and intestinal contents were prepared as follows: 50 μL of the mixed standard working solution of CPT-11, SN-38 and SN-38G at the appropriate concentration and 50 μL of ISTD solution (200 ng/mL) were evaporated to dryness by a gentle stream of nitrogen. Then, a certain volume of the corresponding blank biological matrix was added. The standards and the QC samples for plasma (1, 2, 200, and 800 ng/mL for CPT-11; 0.2, 0.5, 20, and 80 ng/mL for SN-38; and 1, 2, 20, and 80 ng/mL for SN-38G), bile (50, 100, 500, and 2500 ng/mL for CPT-11; 2, 4, 40, and 160 ng/mL for SN-38; and 10, 20, 100, and 250 ng/mL for SN-38G), liver (2, 4, 40, and 160 ng/mL for CPT-11; 1, 2, 20, and 40 ng/mL for SN-38; and 1, 2, 20, and 40 ng/mL for SN-38G), intestinal contents (10, 20, 500, and 1600 ng/mL for CPT-11; 1, 2, 100, and 320 ng/mL for SN-38; and 1, 2, 50, and 160 ng/mL for SN-38G) and intestine (2, 4, 50, and 160 ng/mL for CPT-11; 1, 2, 25, and 80 ng/mL for SN-38; and 1, 2, 25, and 80 ng/mL for SN-38G) were subjected to the proposed pretreatments.

The calibration curves were prepared by plotting the peak area ratio of the analyzed components to the ISTD versus the analyte concentrations using a $1/x$ weighted linear least-square regression model. The linear ranges, regression equations of the curves and correlation coefficients (r) of the plasma, bile, liver, intestine and intestinal contents are listed in **Table S1**.

Precision and Accuracy

The intra-day and inter-day precision (% relative standard deviation, RSD) and accuracy (% relative error, RE) (**Table S2-S6**) were obtained by analyzing the QC samples at four concentration levels (five samples for each concentration level) on the same day and on three consecutive validation days, respectively. The precisions of the QC samples at different concentrations (low, medium and high) in the plasma, bile, liver, intestinal content, and intestine were less than 12.9%, and the accuracies ranged from -10.65% to 11.63%. At the lower limit of quantification (LLOQ), the RSDs were less than 16.13%, and the REs ranged from -9.26% to 16.6%.

Recovery and matrix effect

The extraction recoveries of CPT-11, SN-38 and SN-38G (**Table S2-S6**) at low, medium and high levels from different biological QC samples ranged from 52.02% to 103.49%, with RSD values of less than 14.13%. The matrix effect results for different biological QC samples were 87.13-112.25% for CPT-11, 85.69-113.69% for SN-38,

and 87.79–113.88% for SN-38G, indicating that the matrix effect had a negligible impact on the assay.

Dilution integrity

The dilution integrity was assessed in plasma, bile and intestinal content by diluting five samples with their corresponding blank biological matrixes by factors of ten. The RSDs for the dilution integrity (**Table S7**) were less than 8.14%, and the REs ranged from -7.83% to 7.93%. These data support the use of 10-fold sample dilution for analysis.

Stability

The stabilities of CPT-11, SN-38 and SN-38G (**Table S8**) were evaluated by analyzing the corresponding QC samples at three different concentrations under the following sample storage and processing conditions: storage in an ice bath for 3 h, storage at 20°C for 6 h after being processed, storage at -80°C for one month, and exposure to two freeze-thaw cycles. CPT-11, SN-38 and SN-38G in different biological matrixes were stable according to the stability tests; the accuracy (RE) values ranged from -5.43% to 4.55% for CPT-11, from 1.26% to 10.45% for SN-38 and from -11.96% to -1.93% for SN-38G.

TABLE LEGENDS

Table S1. The Regression data and LLOQs of CPT-11, SN-38 and SN-38G in different biological matrices

Table S2. Recovery, matrix effect, accuracy and precision for CPT-11, SN-38 and SN-38G in plasma (n=5)

Table S3. Recovery, matrix effect, accuracy and precision for CPT-11, SN-38 and SN-38G in bile (n=5)

Table S4. Recovery, matrix effect, accuracy and precision for CPT-11, SN-38 and SN-38G in liver (n=5)

Table S5. Recovery, matrix effect, accuracy and precision for CPT-11, SN-38 and SN-38G in intestinal content (n=5)

Table S6. Recovery, matrix effect, accuracy and precision for CPT-11, SN-38 and SN-38G in intestinal tissue (n=5)

Table S7. The dilution integrity of CPT-11, SN-38 and SN-38G in different biological matrices

Table S8. Stability of CPT-11, SN-38 and SN-38G at different conditions

Table S1. The Regression data and LLOQs of CPT-11, SN-38 and SN-38G in different biological matrices

Sample matrix	Analytes	Calibration curve	Linear range (ng/mL)	*r*	LLOQ (ng/mL)
Plasma	CPT-11	$y = 0.0031x + 0.00183$	1-1000	0.9988	1
	SN-38	$y = 0.0498x + 0.00688$	0.2-100	0.9959	0.2
	SN-38G	$y = 0.00292x + 0.00105$	1-100	0.9991	1
bile	CPT-11	$y = 0.00308x + 0.00954$	50-3200	0.9981	50
	SN-38	$y = 0.0257x + 0.0467$	2-200	0.9956	2
	SN-38G	$y = 0.00629x + 0.000666$	10-320	0.9941	10
liver	CPT-11	$y = 0.00533x - 0.000327$	2-200	0.9979	2
	SN-38	$y = 0.0313x + 0.0134$	1-50	0.9942	1
	SN-38G	y = 0.0123x - 0.00271	1-50	0.9992	1
Intestinal content	CPT-11	$y = 0.00633x + 0.017$	10-2000	0.9988	10
	SN-38	$y = 0.0634x + 0.0958$	1-400	0.9978	1
	SN-38G	$y = 0.0112x + 0.00127$	1-200	0.9994	1
Intestinal tissue	CPT-11	$y = 0.0128x + 0.0421$	2-200	0.9944	2
	SN-38	$y = 0.0411x + 0.0385$	1-100	0.9966	1
	SN-38G	$y = 0.0132x + 0.00225$	1-100	0.9979	1

Table S2. Recovery, matrix effect, accuracy and precision for CPT-11, SN-38 and SN-38G in plasma (n=5)

Compound	Conc.(ng/mL)	Matrix effect		Recovery		Intra-day (n=5)		Inter-day (n=5)	
		Average (%)	RSD (%)	Average (%)	RSD (%)	Precision (RSD, %)	Accuracy (RE, %)	Precision (RSD, %)	Accuracy (RE, %)
CPT-11	1	-	-	-	-	14.53	8.77	10.73	13.35
	2	88.50	5.35	101.26	2.98	10.02	11.63	10.56	11.37
	200	102.14	2.62	91.18	4.61	9.27	-8.65	8.96	8.98
	800	92.18	1.48	103.49	6.10	8.06	8.23	6.53	-8.93
SN-38	0.2	--	-	-	-	12.66	14.86	12.29	13.83
	0.5	113.69	3.78	94.39	2.32	10.14	-10.65	12.88	10.96
	20	110.01	2.17	85.16	9.94	6.22	8.08	9.03	-9.95
	80	111.98	3.26	89.94	2.90	5.63	8.99	9.13	4.69
SN-38G	1	-	-	-	-	12.77	16.60	16.13	13.18
	2	97.43	6.42	66.30	14.09	10.69	-9.93	10.78	-8.33
	20	113.88	8.47	52.02	14.13	11.78	-9.02	9.11	7.65
	80	100.09	4.14	53.62	9.65	9.89	9.22	7.99	-8.03

Table S3. Recovery, matrix effect, accuracy and precision for CPT-11, SN-38 and SN-38G in bile (n=5)

Compound	Conc.(ng/mL)	Matrix effect		Recovery		Intra-day (n=5)		Inter-day (n=5)	
		Average (%)	RSD (%)	Average (%)	RSD (%)	Precision (RSD, %)	Accuracy (RE, %)	Precision (RSD, %)	Accuracy (RE, %)
CPT-11	50	-	-	-	-	8.96	5.67	8.97	12.69
	100	101.66	2.11	90.89	4.95	5.78	4.68	9.26	-6.03
	500	102.25	1.08	88.97	2.93	2.95	-3.69	8.27	3.29
	2500	96.25	2.96	86.39	5.53	2.88	2.67	8.19	-6.22
SN-38	2	-	-	-	-	3.89	1.63	9.01	6.83
	4	94.13	5.05	92.70	2.69	2.69	-4.12	7.29	-5.10
	40	90.69	2.78	90.13	4.55	0.57	1.35	2.93	0.29
	160	89.72	6.88	88.58	2.08	1.56	2.18	2.99	-1.61
SN-38G	10	-	-	-	-	6.78	-5.69	5.98	-7.33
	20	87.79	5.56	89.95	4.16	5.95	4.69	6.75	7.93
	100	88.23	4.96	87.87	5.66	5.89	-6.98	3.78	-4.66
	250	94.29	2.56	85.45	4.68	7.08	8.89	3.56	8.66

Table S4. Recovery, matrix effect, accuracy and precision for CPT-11, SN-38 and SN-38G in liver (n=5)

Compound	Conc.(ng/mL)	Matrix effect		Recovery		Intra-day (n=5)		Inter-day (n=5)	
		Average (%)	RSD (%)	Average (%)	RSD (%)	Precision (RSD, %)	Accuracy (RE, %)	Precision (RSD, %)	Accuracy (RE, %)
CPT-11	2	-	-	-	-	9.27	8.14	8.97	7.55
	4	87.13	5.13	90.89	2.66	7.95	-5.56	6.24	5.10
	40	90.28	2.66	88.22	1.63	4.22	3.11	4.49	-3.61
	160	89.64	3.17	85.73	2.69	1.96	-2.87	3.22	7.80
SN-38	1	-	-	-	-	10.66	8.97	4.69	5.60
	2	86.16	3.27	92.13	4.55	6.43	-7.84	6.03	-5.75
	20	85.69	7.87	89.63	2.10	5.66	7.87	5.13	4.23
	40	92.67	1.69	86.77	3.27	6.54	-7.03	2.26	3.07
SN-38G	1	-	-	-	-	8.89	-9.26	6.78	-8.13
	2	88.10	4.66	76.89	2.66	7.92	8.13	5.74	-5.10
	20	92.17	2.99	74.66	3.77	3.66	-5.24	2.13	1.28
	40	95.33	2.56	73.28	6.63	4.54	3.29	3.69	-4.47

Table S5. Recovery, matrix effect, accuracy and precision for CPT-11, SN-38 and SN-38G in intestinal content (n=5)

Compound	Conc.(ng/mL)	Matrix effect		Recovery		Intra-day (n=5)		Inter-day (n=5)	
		Average (%)	RSD (%)	Average (%)	RSD (%)	Precision (RSD, %)	Accuracy (RE, %)	Precision (RSD, %)	Accuracy (RE, %)
CPT-11	10	-	-	-	-	9.97	5.88	5.69	2.78
	20	88.95	4.25	63.63	1.02	7.67	4.43	6.97	3.08
	500	112.25	4.04	62.33	4.80	5.06	7.12	4.77	1.40
	1600	100.99	2.53	59.79	0.86	6.69	-2.13	6.69	-4.55
SN-38	1	-	-	-	-	12.69	7.92	3.30	2.87
	2	86.48	4.66	72.92	6.72	10.98	-4.63	5.18	-6.98
	100	90.29	3.41	74.39	5.24	9.29	4.03	4.17	9.29
	320	88.78	5.66	74.52	2.96	7.16	-2.09	3.73	-4.98
SN-38G	1	-	-	-	-	7.33	-3.32	2.13	-4.56
	2	89.13	5.19	80.65	4.76	5.77	-4.67	3.66	-4.75
	50	90.12	6.89	62.24	5.83	3.28	6.97	5.77	-4.43
	160	93.29	5.08	65.08	0.58	4.89	-3.19	3.36	4.07

Table S6. Recovery, matrix effect, accuracy and precision for CPT-11, SN-38 and SN-38G in intestinal tissue (n=5)

Compound	Conc.(ng/mL)	Matrix effect		Recovery		Intra-day (n=5)		Inter-day (n=5)	
		Average (%)	RSD (%)	Average (%)	RSD (%)	Precision (RSD, %)	Accuracy (RE, %)	Precision (RSD, %)	Accuracy (RE, %)
CPT-11	2	-	-	-	-	12.77	8.93	6.89	9.22
	4	93.79	1.66	95.22	1.26	9.37	-7.96	5.16	-3.18
	50	92.13	4.37	87.59	5.60	9.85	5.69	4.93	5.67
	160	94.43	4.47	87.58	3.69	7.26	-4.93	5.17	-2.34
SN-38	1	-	-	-	-	10.22	8.69	6.79	8.97
	2	112.28	3.05	84.81	11.72	8.15	-4.47	12.66	-3.06
	25	110.14	7.51	82.34	13.25	1.63	5.66	8.97	-4.65
	80	108.33	6.47	77.36	4.79	6.95	-7.13	6.68	-2.19
SN-38G	1	-	-	-	-	3.89	-4.06	10.09	8.95
	2	111.09	9.81	77.81	8.57	5.77	3.29	8.26	-6.95
	25	100.73	2.45	68.74	1.85	8.16	-2.28	7.13	-2.96
	80	108.88	2.57	68.04	3.71	3.99	4.78	8.18	2.99

Table S7. The dilution integrity of CPT-11, SN-38 and SN-38G in different biological matrices

Sample matrix	Components	RE (%)	RSD (%)
Plasma	CPT-11	1.25	8.14
	SN-38	0.93	4.22
	SN-38G	-3.96	5.92
bile	CPT-11	-6.97	7.82
	SN-38	3.99	4.99
	SN-38G	-2.74	6.28
Intestinal content	CPT-11	7.93	2.61
	SN-38	-1.54	3.97
	SN-38G	-7.83	5.38

Table S8. Stability of CPT-11, SN-38 and SN-38G at different conditions

Sample matrix	Components	Nominal conc. (ng/mL)	in ice bath for 3 h	at 20˚C for 6 h after processed	at −80˚C for one month	two freeze–thaw cycles
			Accuracy (RE, %)	Accuracy (RE, %)	Accuracy (RE, %)	Accuracy (RE, %)
Plasma	CPT-11	2	-4.69	-2.37	-2.97	-3.16
		200	-3.23	-4.98	-1.90	-4.91
		800	0.69	-1.78	-2.11	-3.29
	SN-38	0.5	1.84	3.76	2.48	3.56
		20	2.04	2.34	3.91	4.30
		80	1.26	4.66	2.56	2.09
	SN-38G	2	-8.97	-6.93	-5.66	-9.28
		20	-5.64	-5.86	-6.93	-6.42
		80	-3.57	-4.97	-4.53	-5.75
bile	CPT-11	100	0.34	0.65	3.19	2.07
		500	1.06	1.22	2.05	1.33
		2500	1.39	4.55	0.66	0.49
	SN-38	4	3.49	2.75	4.39	3.09
		40	1.66	4.56	3.22	2.93
		160	1.53	2.89	4.11	2.13
	SN-38G	20	-2.54	-3.69	-2.17	-3.14
		100	-3.51	-2.57	-1.78	-2.92
		250	-1.63	-2.13	-2.93	-1.74
liver	CPT-11	4	-0.69	-2.45	-4.37	-2.08
		40	-1.05	-3.19	-3.29	-3.66
		160	-1.28	-2.67	-1.46	-2.29
	SN-38	2	6.77	5.78	5.36	3.94
		20	5.43	4.85	5.29	3.05
		40	4.32	5.94	4.05	2.03
	SN-38G	2	-4.87	-4.65	-5.32	-4.29
		20	-3.95	-5.59	-4.95	-6.93
		40	-3.47	-4.85	-5.37	-4.97

Table S8 (*Continued*)

Sample matrix	Components	Nominal conc. (ng/mL)	in ice bath for 3 h	at 20˚C for 6 h after processed	at −80˚C for one month	two freeze–thaw cycles
Intestinal content	CPT-11	20	1.54	2.67	2.56	3.43
		500	1.33	3.04	3.04	4.27
		1600	1.28	2.15	2.76	3.83
	SN-38	2	6.67	9.46	6.64	8.69
		100	6.93	10.45	7.98	7.32
		320	7.36	8.67	4.39	2.89
	SN-38G	2	-10.45	-7.38	-4.26	-11.96
		50	-9.77	-5.67	-3.17	-8.77
		160	-10.49	-4.49	-4.94	-7.29
Intestinal tissue	CPT-11	4	-3.88	-2.67	-3.27	-2.45
		50	-4.59	-3.84	-2.45	-3.29
		160	-5.43	-2.29	-2.29	-2.37
	SN-38	2	4.98	2.27	7.29	4.57
		25	3.26	3.16	8.63	2.39
		80	3.19	5.68	3.99	3.95
	SN-38G	2	-4.96	-6.93	-3.97	-8.65
		25	-7.33	-7.87	-4.29	-9.79
		80	-5.26	-9.45	-4.61	-8.97
